# Episodic memory involves transient and sparse connectivity aligned to both internal and external events

**DOI:** 10.1371/journal.pbio.3003481

**Published:** 2025-11-25

**Authors:** Adam J. O. Dede, Zachariah R. Cross, Samantha M. Gray, Joseph P. Kelly, Qin Yin, Parisa Vahidi, Eishi Asano, Stephan U. Schuele, Joshua M. Rosenow, Joyce Y. Wu, Sandi K. Lam, Jeffrey S. Raskin, Jack J. Lin, Olivia Kim McManus, Shifteh Sattar, Ammar Shaikhouni, David King-Stephens, Peter B. Weber, Kenneth D. Laxer, Peter Brunner, Jarod L. Roland, Ignacio Saez, Fady Girgis, Robert T. Knight, Noa Ofen, Elizabeth L. Johnson

**Affiliations:** 1 Northwestern University, Evanston, Illinois, United States of America; 2 Stanford University, Stanford, California, United States of America; 3 University of Texas at Dallas, Richardson, Texas, United States of America; 4 Wayne State University, Detroit, Michigan, United States of America; 5 The Ann & Robert H. Lurie Children’s Hospital of Chicago, Chicago, Illinois, United States of America; 6 University of California, Davis, California, United States of America; 7 University of California, San Diego, California, United States of America; 8 UCSD Rady Children’s Hospital, San Diego, California, United States of America; 9 Ohio State University and Nationwide Children’s Hospital, Columbus, Ohio, United States of America; 10 California Pacific Medical Center, San Francisco, California, United States of America; 11 Yale University, New Haven, Connecticut, United States of America; 12 Washington University in St Louis, St. Louis, Missouri, United States of America; 13 Ichan School of Medicine at Mount Sinai, New York, New York, United States of America; 14 University of Calgary, Calgary, Canada; 15 University of California, Berkeley, California, United States of America; University of Glasgow, UNITED KINGDOM OF GREAT BRITAIN AND NORTHERN IRELAND

## Abstract

Episodic memory depends on the coordination of local processing, indexed by high-frequency broadband (HFB) activity, with global organization, indexed by theta oscillations. However, theta and HFB exhibit asynchronous timing, raising the question of how results of local processing are communicated. Using intracranial EEG in patients performing a recognition memory task, we examined this coordination across medial temporal (MTL) and prefrontal (PFC) regions. HFB peaks occurred earlier in the MTL than in the PFC. Contrasting analyses were anchored either to these internally driven HFB peaks or to the external event of stimulus presentation. We discovered three key results. First, the role of the PFC changed from encoding to retrieval. Specifically, PFC-MTL theta connectivity was aligned with internal PFC peaks during encoding, suggesting top-down initiation. By contrast, this connection was aligned with external stimulus presentation during retrieval, suggesting bottom-up initiation. Second, the anterior cingulate cortex exhibited connectivity that was aligned to internal HFB peaks only, suggesting that its role is evaluative, devoid of direct stimulus processing. Third, graph theoretic analysis of whole-brain connectivity patterns revealed that the connections predicting successful memory performance were embedded in transient, sparse network states. These results reveal that analyses triggered from internally-generated events yield different results when compared to classic analyses triggered using external events. The picture that emerges is a sequence of specific, short-lived, internally-generated states that drive episodic memory success.

## Abstract 2

Episodic memory depends on the coordination of local processing with global organization. This study reveals that episodic memory relies on brief, sparsely connected brain states coordinated across medial temporal and prefrontal regions, uncovering dynamic shifts in network directionality and timing between encoding and retrieval.

## Introduction

High-frequency EEG components reflect local processing [1–5], while low-frequency components support network organization and information flow [6]. The coordination of local and network-level signals is essential for complex processes like episodic memory, and both theta (2–8 Hz) and high-frequency broadband (HFB; 70–150 Hz) signals have been extensively studied during memory tasks [7–20]. Precisely how these signals interact during successful episodic memory in humans—especially in time—remains unclear.

Intracranial EEG (iEEG) studies show that HFB activity peaks at different times across brain regions during memory encoding, with latencies progressing along the ventral visual pathway to the medial temporal lobe (MTL) and later to association regions including the prefrontal cortex (PFC) [7–10,12]. PFC activity occurs earlier during retrieval [7,8,11]. These findings suggest that complex processing occurs through sequences of transient states [21–23]. By contrast, theta activity spans the brain shortly after stimulus onset [9,24], often out of sync with HFB activity [10,13–20]. This raises a central question: if theta reflects global organization but does not coincide with local HFB processing, how are results of local processing shared across the network?

The method of trial alignment may play a critical role in understanding this interaction. Peaks in HFB activity exhibit inter-trial variability [25], are brief [26–28], and resemble point processes similar to neuronal bursts [29,30]. If local processing is transient, then analyses of theta activity should use short temporal windows and consider effects that are temporally aligned to both internally driven HFB peaks and traditional external variables such as stimulus onset. Trial-averaging long epochs aligned exclusively to external events may obscure important aspects of theta dynamics [23].

We hypothesized that aspects of network modulation in the theta band would be locked to internally-generated HFB peaks. Because the timing of HFB peaks exhibits high variability from one trial to another, traditional analyses are blind to theta modulation in response to these internal events. The key innovation of this study was to compare results based on aligning data to internal timing of local HFB peaks (HFB-locked) to results based on aligning data to external timing of stimulus onset (image-locked). We predicted that our approach would reveal different patterns of results as a function of time alignment, demonstrating the importance of both internal and external events during mnemonic processing. To test this, we analyzed iEEG data from epilepsy patients performing an established memory task [31–35], focusing on five regions of interest (ROIs): hippocampus (Hip), parahippocampal gyrus (PHG), anterior cingulate cortex (ACC), dorsolateral prefrontal cortex (dlPFC), and polar prefrontal cortex (pPFC). We first confirmed that HFB peak latencies followed the previously described MTL-to-PFC temporal cascade [7,9,13]. We then examined how theta signal organization related to memory success, assessing both local phase clustering and between-region connectivity in both HFB-locked and image-locked analyses. Supporting our hypothesis, our results converge to suggest that sequences of transient, sparse, internally-generated network states drive episodic memory success.

## Results

**Fig 1:**
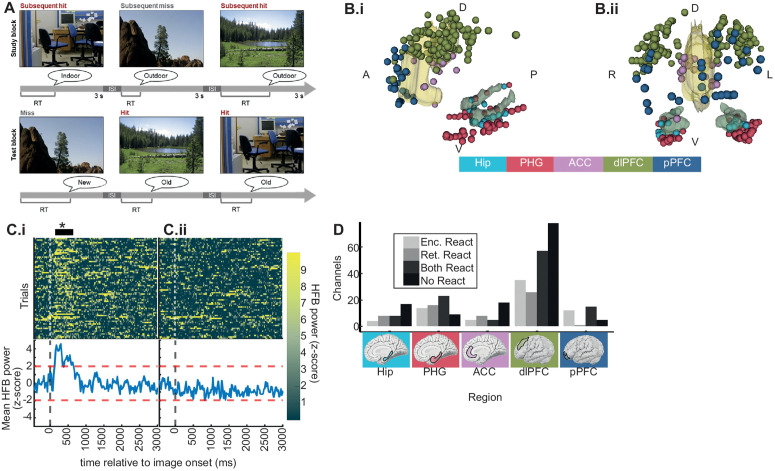
Task and channel coverage. **A.** Schematic of the behavioral task. During study blocks, participants made indoor/outdoor judgements while viewing images of natural scenes. During test blocks, participants made old/new judgements while viewing the same images of natural scenes intermixed with novel images. All scene images were taken by author Noa Ofen and are reproduced here with permission. **B.** Channel coverage combined across participants. i. Lateral view. ii. Face-on view. ACC and Hip ROIs are shown with 3D models for reference. **C.** Example channels demonstrating detection of reactive channels. Top panels display heatmaps with trials on the *y* axis, time relative to image onset on the *x* axis, and the HFB power indicated by color. Bottom panels display the mean HFB power across trials on the *y* axis and time on the *x* axis. Notice that in panel i, there is a response soon after image onset that exceeds the threshold of z > 2 for more than 50 continuous ms (black horizontal line marked by asterisk above panel). By contrast, in panel ii, the mean response does not exceed the z-score threshold. Example channels were drawn from within a single participant’s PHG, demonstrating within region heterogeneity. This panel can be regenerated using data contained in HFB_singleTrialmtl_sub_image.mat and code in Figure 1C_2A.m [112]. **D.** Summary of reactive and non-reactive channel counts across all ROIs. This panel can be regenerated using data contained in trialLatDat_RTfix.csv and code in Figure1D_encRet.Rmd [112].

Thirty-six participants performed an image recognition memory task (Fig 1A) [31–35]. The age, sex, and memory performance of participants did not vary by electrode coverage across five ROIs (S1 Table; Fig 1B). To relate signals at lower frequencies to peak HFB activity, channels were selected within ROIs that exhibited task-reactive increases in HFB activity (see Star methods; Fig 1C) [31,36–40]. The proportion of channels exhibiting reactivity varied between regions ${(\chi }^{\text{2}}(\text{4})=\text{1}\text{9},p=.\text{0}\text{0}\text{0}\text{9})$ (Fig 1D). All subsequent analyses utilized only channels identified as reactive.

### Latency of HFB activity replicates known sequence

This analysis focused on the latency of HFB peaks. A parallel analysis of HFB power is presented in S1 Fig.

**Fig 2:**
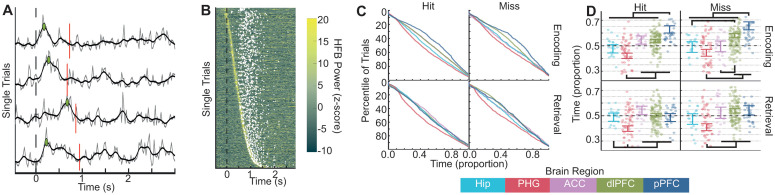
High-frequency broadband (HFB) peak latency timing changes with memory. **A.** In grey, four sequentially recorded examples of single-trial HFB traces are displayed from a single channel. In black, the same traces are displayed after application of a Gaussian smoothing kernel. The broken vertical red line indicates the behavioral RT of each trial. The dashed vertical black line indicates image onset. The green dots indicate the peak HFB activity on each trial. Note the lack of consistency in peak latency. This panel can be regenerated using data contained in HFB_singleTrialmtl_sub_image.mat and code in Figure1C_2A.m [112]. **B.** The heatmap displays single-trial HFB time series from channels in the hippocampus during subsequent hit trials. Trials have been sorted by latency of the peak HFB power. White dots indicate the behavioral RT of each trial, which was a poor predictor of the latency of the HFB power peak. This panel can be regenerated using data contained in HFB_singleTrialmtl_sub_image.mat and code in Figure2B.m [112]. **C.** Each cumulative distribution plot displays the timing of the latency of peak HFB activity across all trials for subsequent hits (top left), subsequent misses (top right), retrieval hits (bottom left), and retrieval misses (bottom right). Each region’s trial distribution is shown with a different line. Because different numbers of trials were observed in different behavioral conditions and for different regions, trial is plotted as a percentile of trials on the *y*-axis. Because reaction times were variable between individuals and conditions, time is plotted on the *x*-axis as a proportion of time such that 0 is image onset and 1.0 is behavioral response. This panel can be regenerated using data contained in the HFB_singleTrial folder and code in Figure2C.m [112]. **D.** Each grouped scatter plot displays the mean time of peak HFB latency for each channel grouped by region. Time relative to image onset is displayed as a proportion on the *y*-axis. Error bars display the 83% confidence interval around model estimates [113,114]. For example, note that while the hippocampus (light blue) and parahippocampal gyrus (red) led the dlPFC and pPFC during successful encoding, these PFC regions were active simultaneously with the Hip during successful retrieval. This panel can be regenerated using data contained in trialLatDat_RTfix.csv and code in Latency_LME_modeling.Rmd [112].

We calculated the latency of HFB peak activity between image onset and behavioral reaction time (RT) for each trial on each channel (Fig 2A–2C). Because peak latencies were detected between image onset and RT, they were biased to exhibit a relationship with RT (correlation between HFB peak latency and RT: r=.45,p<2e−16,t(40281)=102). That is, if it is the case that local HFB peaks form a sequence of activity, then this sequence may sometimes occur at a faster or slower rate without altering the sequence. This possibility is consistent with the observed HFB peak latency and RT correlation. Critically, if this is the case, then the sequence is not fundamentally altered on faster versus slower trials. To adjust for this, we divided peak latencies by RTs and observed no relationship between normalized HFB peak latency and RT (r=−.008,p=.1,t(40281)=−1.6). Thus, the relative timing of HFB peaks did not vary significantly with behavior.

Linear mixed effects modeling of latency as a function of hit/miss by encode/retrieve by region revealed different temporal dynamics in different regions and as a function of mnemonic success during encoding versus retrieval (Fig 2D). Specifically, there were main effects of hit/miss ${(\chi }^{\text{2}}(\text{1})=\text{1}\text{7},p=.\text{0}\text{0}\text{0}\text{0}\text{3})$, encode/retrieve ${(\chi }^{\text{2}}(\text{1})=\text{4}\text{8},p=\text{4}e-\text{1}\text{2})$, and region ${(\chi }^{\text{2}}(\text{4})=\text{7}\text{2},p=\text{8}e-\text{1}\text{5})$, and the interactions were significant between hit/miss and region ${(\chi }^{\text{2}}(\text{4})=\text{1}\text{4},p=.\text{0}\text{0}\text{7})$ and between encode/retrieve and region ${(\chi }^{\text{2}}(\text{4})=\text{4}\text{7},p=\text{2}e-\text{9})$.

During successful encoding (hit trials), the PHG exhibited an HFB peak that occurred earlier than in all three PFC ROIs, with the pPFC latency occurring later than in all other ROIs. During failed encoding (miss trials), MTL ROIs were co-active with the ACC, which shifted earlier, and the dlPFC latency occurred later. HFB peaks in the ACC and PHG also exhibited mnemonic changes in power during encoding (S1C Fig).

During retrieval of studied scenes (hit trials), the PHG exhibited the earliest peak, and the other four ROIs exhibited latencies similar to each other. This pattern was similar for misses, although both the dlPFC and pPFC exhibited significantly delayed latencies during miss trials relative to hits. Because it is possible that these timing effects were driven by individual differences between participants with coverage of different ROIs, we confirmed this analysis in several alternate ways (S2 Fig). These analyses yielded qualitatively similar results.

Taken together, these results replicate previous reports of the sequence of activity underlying episodic memory [7,9,13] and how it changes as a function of encoding/retrieval and memory success. Unlike the separation between MTL and PFC ROIs during successful encoding, HFB activity during hit retrieval trials still peaks first in the PHG, but then the Hip and PFC ROIs all exhibited similar peak latencies. When memory failed, these timing effects were altered.

### Mnemonic differences in theta phase clustering depend on time alignment

It is well known that theta oscillations exhibit phase clustering temporally aligned to stimulus events [41–44], and we reasoned that HFB peaks would act similarly as temporal anchors for network organization. Just as stimulus information must be transmitted to the broader network through low-frequency mechanisms, so too must the results of local processing be transmitted onwards. We compared encoding and retrieval trials sorted by memory outcome (subsequent hit, subsequent miss, hit, miss) using theta phase clustering (inter-trial phase clustering [ITPC]; see Methods), a measure of the consistency of oscillatory phase at a particular time point across trials. This analysis was performed twice for each ROI: HFB-locked and image-locked. In this way, it was possible to compare how signals were organized relative to the internal event of peak local activity and relative to the external event of stimulus presentation. We focused on phase-based measurements to capture the fine-grained temporal organization of low-frequency oscillations. A parallel analysis of low-frequency power is presented in S3 Fig.

**Fig 3:**
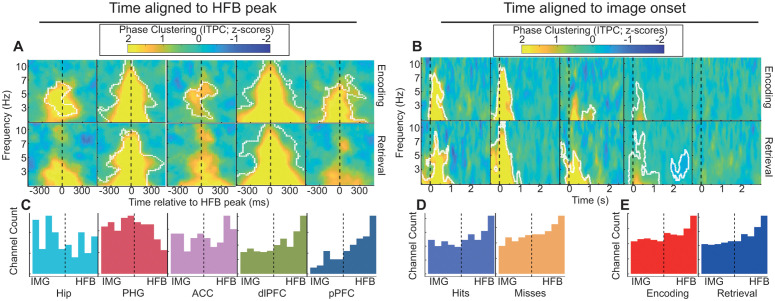
Patterns of mnemonic differences in phase clustering are dependent on time alignment. **A.** Heatmaps display the mean difference in z-scored ITPC between hit and miss trials. The x-axis displays time in milliseconds relative to HFB peak, and the y-axis displays frequency. Color indicates the difference between (subsequent) hit and miss trials in units of z-scored ITPC. Encoding and retrieval data are plotted along the top and bottom rows, respectively. White outlines indicate statistical significance after permutation-based cluster correction. This panel can be regenerated using data contained in the TFphase_HFB folder and code in Figure3A_heatmapVersion.m [112]. **B.** Similar to A except the x-axis displays time in seconds relative to image onset. Note that the dlPFC and pPFC exhibited strong hit/miss phase clustering effects when analysis was locked to local HFB peaks (panel A), but phase clustering effects are weaker when analysis is locked to image onset. This panel can be regenerated using data contained in the TFphase_image folder and code in Figure3B_supFigure4B.m [112]. **C.** Histograms display the IMG-HFB index for different regions collapsed across hit/miss and encode/retrieve trial types. Values at the extremes indicated that phase clustering was observed exclusively when data were analyzed relative to image onset (left) or HFB peak (right). Intermediate values (near the vertical dashed line) indicated that the two treatments of time yielded similar results. Note that the distribution of channels in the dlPFC and pPFC is strongly skewed to the HFB end of the axis. This panel can be regenerated using data **D.**contained in HFB_IMG_index2.csv and code in HFB_IMG_index.Rmd lines 129–169 [112]. **D.** The same as panel C but with histograms representing hit and miss trials collapsed across region and encoding/retrieval. This panel can be regenerated using data contained in HFB_IMG_index2.csv and code in HFB_IMG_index.Rmd lines 172–207 [112]. **E.** The same as panel C but with histograms representing encoding and retrieval trials collapsed across region and hit/miss. The high channel count in the dlPFC leads to this region dominating the distribution shape in panels D and E, but the key observation is that IMG-HFB index values do not vary with hit/miss or encode/retrieve. This panel can be regenerated using data contained in HFB_IMG_index2.csv and code in HFB_IMG_index.Rmd lines 213–252 [112].

In the HFB-locked analysis, phase clustering was stronger for subsequent hit and hit trials over subsequent miss and miss trials (Fig 3A). Specifically, we observed greater phase clustering for subsequent-hit compared to subsequent-miss trials across frequencies concentrated in the theta range in all five ROIs, as well as greater phase clustering for hit compared to miss trials at retrieval in the PHG and dlPFC (all cluster-corrected *p* < .05). These results indicate that increased phase clustering time-locked to HFB peaks is an important organizing principle of signals associated with successful memory. For image-locked ITPC analyses, there appeared to be a gradient across ROIs such that the MTL ROIs exhibited strong phase clustering across 2 to 10 Hz between −75 and 825 ms that differed between (subsequent) hit and miss trials, while this effect became weaker in the ACC, yet weaker in the dlPFC, and non-existent in the pPFC (Fig 3B).

To quantify the relative strength of image- versus HFB-locked ITPC, we calculated a normalized IMG-HFB index score for each channel (see Methods; Fig 3C-E). This score could range from −1 to +1, where +1 indicated that phase clustering was much stronger for the HFB-locked over the image-locked analysis. When IMG-HFB scores were modeled as a function of hit/miss, encode/retrieve, and region, only the main effect of region was significant $\left(\chi^{\text{2}}(\text{4})=\text{3}\text{1},p=\text{3}e-\text{6}\right)$. Visual inspection of Fig 3C revealed that the dlPFC and pPFC exhibited stronger HFB-locked phase clustering, whereas IMG-HFB scores in other regions were more balanced.

Taken together this analysis shows that the Hip participates more strongly in stimulus-driven mnemonic processing, exhibiting stronger hit/miss differences in phase clustering in the image-locked analysis. By contrast, processing in the dlPFC and pPFC appears to be organized around internal events. The PHG and ACC appear to participate in both. Interestingly, although the Hip and PHG exhibited strong phase clustering in both image- and HFB-locked analyses, these regions exhibited markedly higher phase angle consistency across channels in the HFB-locked analysis (compare S4A–S4B Fig). This implies that network organization across channels was more consistent when data were aligned with HFB peaks than when data were aligned with image onset. These results support the idea that transient internal events are strong temporal anchors for network organization similar to external stimulus events, and the PFC is more strongly influenced by internal events while the MTL is more strongly influenced by external events.

Only in the Hip and ACC were significant clusters of ITPC differences between hit and miss trials non-continuous with 2 Hz, the bottom end of our analysis range. This raised the possibility that significant differences in ITPC in other regions may have reflected singular deflections as opposed to sustained theta oscillatory alignment. However, at least three other aspects of the results suggest that true theta oscillatory modulation did occur at the time of HFB peaks. First, low-frequency (2–80 Hz) power spectra derived from HFB-locked data exhibited peaks that were higher than 2 Hz (S3A to S3C Fig; S1 Table). Second, many of the HFB-locked mnemonic differences in connectivity were at frequencies above 2 Hz, which would be unexpected if there were simply a widespread singular deflection. Third, if ITPC effects were driven by broadband deflections in the EEG, then it would be expected that higher HFB power would be associated with higher ITPC strengths. In all cases HFB-locked ITPC was higher for hits over misses, yet HFB peak amplitude was higher for misses than for hits (S1B Fig).

### Mnemonic differences in theta connectivity depend on time alignment

We next asked two key connectivity questions. First, given that temporal patterns of HFB peaks and theta clustering changed between encoding and retrieval, would inter-regional connectivity also change in its timing between encoding and retrieval, particularly the long-distance connections between the PFC and MTL? Second, just as theta clustering effects were different when aligned to HFB peaks versus image onset, would inter-regional connectivity also exhibit different patterns to internal versus external temporal alignment? To understand how connectivity between ROIs at low frequencies varied as a function of memory, we compared connectivity (pairwise phase consistency [PPC]; see Methods) [45] at encoding and retrieval trials sorted by memory outcome independently for each pair of channels. As with phase clustering, this analysis was performed with both image- and HFB-locked data. This analysis was sensitive to the fine time scale organization of low-frequency networks because our measure of connectivity is based on the consistency of the phase offset between channels at a particular time point across trials.

**Fig 4:**
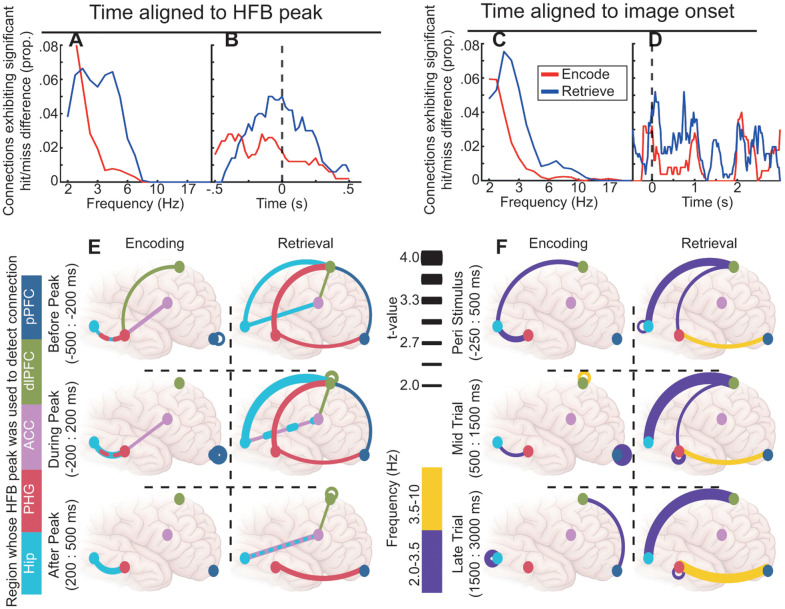
Patterns of connectivity were different depending on time alignment. In panels A-D, the Y axes display the proportion of the time-frequency space for all tested connections that exhibited a significant difference between hit and miss trials after permutation-based cluster correction. **A.** The *x*-axis displays frequency. **B.** The *x*-axis displays time relative to HFB peak. **C.** As A for image-locked analysis. **D.** The *x*-axis displays time relative to image onset. Panels A,B,C, and D can be regenerated using data contained in HFBConnections.mat and imageConnections.mat and code in Figure4ABCD.m [112]. **E.** The connection schematics display connections that were significantly increased for hit relative to miss trials for HFB-locked analysis. Line colors indicate the region whose HFB peak was used for temporal alignment. The rows display those connections that exhibited significance before (top), during (middle), and after (bottom) the HFB peak. The columns correspond to encoding (left) and retrieval (right). Dots represent color-coded region locations. The thickness of lines connecting dots represents the t-value of the difference between hit and miss trial connectivity. Connections between pairs of channels within a single ROI are represented with self-referencing loops. **F.** Line colors indicate the mean frequency of the significant difference between hit and miss trials. The connection schematics reflect mnemonic connections from the image-locked analysis. All other conventions are similar to E except that time is divided into peri-stimulus (top), mid-trial (middle), and late-trial (bottom) periods. Panels E and F can be regenerated using data contained in HFBConnections.mat and imageConnections.mat and code in Figure4EF.m [112]. For hit less than miss differences, see S7 Fig.

Many pairwise connections differed (*p* < .05) after cluster correction for multiple comparisons across all time-frequency points that were tested (see Methods) based on memory outcome (hereafter termed mnemonic connections), but these were not evenly distributed across encoding/retrieval, frequency, time, region, or analysis approach. In general, there were more mnemonic connections during retrieval than during encoding, and 72% of all mnemonic connections were increases in connectivity for positive memory outcomes (subsequent hits, hits) over negative memory outcomes (subsequent misses, misses). We focus on these positive mnemonic connections; see S5 Fig for a schematic representation of negative mnemonic connections. To descriptively represent patterns in the time-frequency extent of significant clusters, we calculated the average proportion of time points with significant mnemonic differences across all connections for each frequency (Fig 4A and 4C), and for each time point (Fig 4B and 4D). Mnemonic connections were concentrated in frequencies below 10 Hz (Fig 4A and 4C), with encoding effects largely below 3.5 Hz and retrieval effects ranging up to 10 Hz. The timing of mnemonic connections varied between encoding and retrieval. Specifically, HFB-locked mnemonic connections occurred prior to HFB peaks during encoding, but were centered around HFB peaks during retrieval (Fig 4B). Image-locked mnemonic connections were most prevalent around image onset and late in the trial during encoding, but were observed across the entire trial during retrieval (Fig 4D). For visualization, mnemonic connections are indicated schematically in Fig 4E and 4F; time-frequency representations of connectivity are presented in S6-S9 Figs.

HFB-locked analysis of encoding revealed mnemonic connections between the Hip and PHG, dlPFC and PHG, and ACC and PHG (Fig 4E). For HFB-locked analysis of connectivity between any two ROIs, one ROI’s HFB peak had to be used for temporal alignment of both ROIs. For all pairwise connections, this analysis was repeated twice in order to use each ROI’s HFB peak for temporal alignment. Results often differed markedly depending on which ROI’s HFB peak was used. The ROI whose HFB peak was used for temporal alignment in the detection of significant mnemonic connections is represented by the color of the line connecting the ROI’s in Fig 4E. Consideration of which ROI’s HFB peak had been used to detect each mnemonic connection, combined with the timing of HFB peaks (see Fig 2D), suggested a dynamic sequence of connectivity patterns. Specifically, successful encoding was marked by connectivity within the MTL that spanned both ROI’s local HFB peaks, followed by connectivity between the PHG and dlPFC/ACC that preceded the HFB peaks in PFC ROIs. Analysis of retrieval revealed mnemonic connections between the MTL ROIs and all three PFC ROIs, but not within the MTL. Long-distance mnemonic connections between the MTL and PFC were associated with the timing of PFC HFB peaks during encoding and with the timing of MTL peaks during retrieval, suggesting a reversal of connectivity direction. This reversal highlights a shift from top-down, internally driven coordination during encoding to early, bottom-up signaling during retrieval. However, there were still mnemonic connections within the PFC locked to HFB peaks within the PFC (Figs 4E and S9), suggesting potential continued prefrontal processing after initial connectivity with the MTL during retrieval.

Image-locked analysis of encoding revealed mnemonic connections between the Hip and dlPFC prior to image onset, within the MTL throughout the trial, and between the pPFC and dlPFC late in the trial (Fig 4F). Notably absent were connections between the MTL and PFC after image onset. Analysis of retrieval revealed a more extensive set of mnemonic connections. In particular, connections between the PFC and MTL were observed throughout the trial. Interestingly, mnemonic connections involving the ACC were absent in image-locked analyses, supporting the conclusion that the ACC’s role in memory is primarily internally coordinated and may not be fully detected by traditional stimulus-locked approaches..

Taken together, mnemonic connections between MTL and PFC ROIs were more prevalent during retrieval than encoding. The MTL-PFC connections that were identified during encoding were aligned to the HFB peaks of PFC ROIs, suggesting that these late connections may be recruited by local activity in the PFC. By contrast, mnemonic connections during retrieval were more likely to be aligned to the HFB peaks of MTL ROIs, or in the case of image-locked connections, persisted throughout the trial. These results, together with temporal alignment between Hip and PFC HFB peak latencies (see Fig 2D), suggest early integration between the MTL and PFC to facilitate retrieval. Finally, mnemonic connections involving the ACC were only detectable in the HFB-locked analysis for both encoding and retrieval.

### Mnemonic increases in theta connectivity are contextualized by sparse network states

In both HFB- and image-locked analyses, we detected short periods of mnemonic modulation of connectivity. We next tested whether increases in connectivity between ROIs occurred in the context of globally elevated connectivity at these specific moments in time, similar to the findings reported by prior studies using longer analysis windows [46,47], or whether they reflected selective tuning. To address this issue, for each significant change in ROI-ROI connectivity, each participant’s full channel X channel connectivity matrix was extracted for the time and frequency ranges of the significant difference. This was done separately for different trial types (subsequent hit, subsequent miss, hit, miss). For example, in the HFB-locked analysis of retrieval, hit trials were characterized by a significant increase in connectivity between the Hip and ACC concentrated in low frequencies after the ACC HFB peak (S9 Fig). All six participants with electrodes in both of these regions exhibited this difference (S10A Fig).

**Fig 5:**
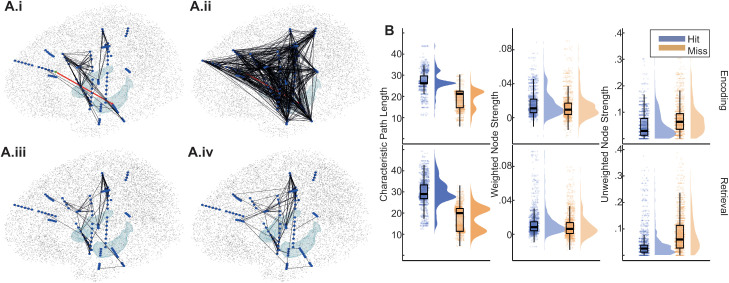
Short periods of increased connectivity between ROIs are characterized by a sparse overall network state. **A**. Plots display binarized connectivity graphs for a single participant between 2.6 and 6.6 Hz during retrieval. Blue dots indicate recording channels. Light blue surfaces indicate the location of the hippocampi for anatomical reference. Black stippling indicates the cortical surface. Black lines represent connections with suprathreshold connectivity strength (pairwise phase consistency). i and ii display connections averaged across the time window between −425 and −75 ms relative to the Hip HFB peak. The red line indicates a connection between the ACC and Hip, which was elevated during successful retrieval for all participants (S10A Fig). Panel i displays connections during hit trials. ii. displays connections during miss trials. The binarization threshold was .1. This yielded 374 suprathreshold connections during hit trials and 1,622 during miss trials. iii and iv display similar connection graphs with connectivity values averaged across the time window between 0 and 1,500 ms relative to image onset. The binarization threshold was adjusted to .0686, which yielded 374 suprathreshold connections during hit trials—the same number as observed in the short time window analysis. However, unlike the short-time window analysis, there was not a marked increase in suprathreshold connections during miss trials. Specifically, there were 440 suprathreshold connections during miss trials. This panel can be regenerated using data contained in hip_acc_ret_HFB_2_21.mat and code in Figure5A_supFigure10B.m [112]. **B.** Graph theoretic measures taken for all connectivity graphs calculated using the time-frequency parameters of significant hit v. miss clusters. Characteristic path length and unweighted node strength both indicated more connected graphs during miss trials than hit trials, extending the effects seen in A across all participants and all connections. Each point represents one combination of participant, analysis timing (HFB-locked vs. image-locked), time, frequency, region 1, and region 2. Plots displaying these data separated by region and analysis timing are presented in S10 Fig. This panel can be regenerated using data contained in graphDat.csv and code in graphMeasures.Rmd lines 336–432 [112].

The connectivity matrices averaged across significant time and frequency ranges for a single representative participant were binarized and plotted in MNI anatomical space for visualization (Fig 5A.i and 5A.ii). Although the time and frequency ranges used to extract these values were chosen because of greater hit-over-miss connectivity between ROIs, visual inspection suggested that the pattern of connectivity across all channels was one of greater connectivity during miss trials. By contrast, when connectivity values were extracted at the same frequencies but averaged across a larger time window (between 0 and 1.5 seconds after image onset [47]), there was little difference between the connectivity patterns observed for hit compared to miss trials (Fig 5A.iii and 5A.iv). Specifically, for all panels in Fig 5A, connectivity matrices were binarized for visualization. The binarization threshold in panels A.i and A.ii was PPC = .1. This yielded 374 suprathreshold connections for hit trials (Fig 5A.i) and 1,622 for miss trials, a more than 4-fold increase. For panels A.iii and A.iv, the binarization threshold was adjusted to PPC = .0686. This yielded 374 suprathreshold connections for hit trials, matching the number observed in the short time window analysis. However, only 440 connections exhibited suprathreshold connectivity during miss trials, a much smaller increase than was observed in the short time window analysis. Thus, for this example participant, the transient increase in Hip-ACC connectivity observed during successful retrieval appears to have been specifically important for memory success as opposed to being a random sample from a network with high levels of connectivity everywhere. The contrast of increased connectivity between ROIs with decreased connectivity across the brain may be a marker of increased network tuning to facilitate memory success.

To evaluate network tuning across all participants and all connections, graph theoretic analysis was applied to the channel X channel connectivity matrices for each participant at the time and frequency range of each significant change in connectivity detected between ROIs. Graph theory provides descriptive measures capable of quantifying the overall connectivity state of a network [48]. Each significant change in connectivity was characterized by a particular analysis timing (HFB-locked versus image-locked), time, frequency, region 1, and region 2. For statistical analysis, graph measures were combined across levels of time, frequency, and region 2. Graph measures were analyzed using linear mixed effects modeling as a function of memory outcome (subsequent hit/subsequent miss, or hit/miss) by region 1 by analysis timing. These analyses were repeated for encoding and retrieval separately. Thus, this analysis asked, what was the overall network state associated with mnemonic connections detected for each region and analysis timing separately? Three graph measures were calculated [48]. The characteristic path length is the average distance needed to traverse the graph between any two channels. The weighted strength is the summed connectivity values across all channels paired with a single seed channel in region 1. The unweighted strength is the proportion of values greater than.1 across all channels paired with a single seed channel in region 1.

Across encoding and retrieval, there were main effects of memory outcome such that characteristic path length was longer and unweighted strength was lower during hit relative to miss trials $\left(\chi^{\text{2}}(\text{1})>\text{7}\text{8}\text{8},maximump<\text{2}e-\text{1}\text{6}\right)$ (Fig 5B). Although weighted strength was higher for hit than for miss trials $\left(\chi^{\text{2}}(\text{1})>\text{1}\text{1}\text{3},p<\text{2}e-\text{1}\text{6}\right)$, the size of this effect was smaller than observed for the other two measures. These results echo the intuition gained from visual examination of Fig 5A.i and 5A.ii. That is, when specific ROI-ROI connections increased during successful memory outcomes, the network became more specifically tuned, resulting in longer characteristic path lengths, and the proportion of connections exhibiting high strength decreased (see S10 Fig for results separated by ROI).

Taken together, these results demonstrate that increased connectivity between ROIs in association with successful memory was accompanied by decreased connectivity throughout the brain in general. Specific connectivity effects reflected transient and discrete temporal epochs, and this was true for both HFB- and image-locked connections. These results challenge prior work based on long, stimulus-locked temporal epochs in which broadly elevated connectivity during memory has been revealed, and instead support a model of transient, targeted coordination.

### Phase clustering and connectivity within and between the MTL and dlPFC predict individual memory performance

**Fig 6:**
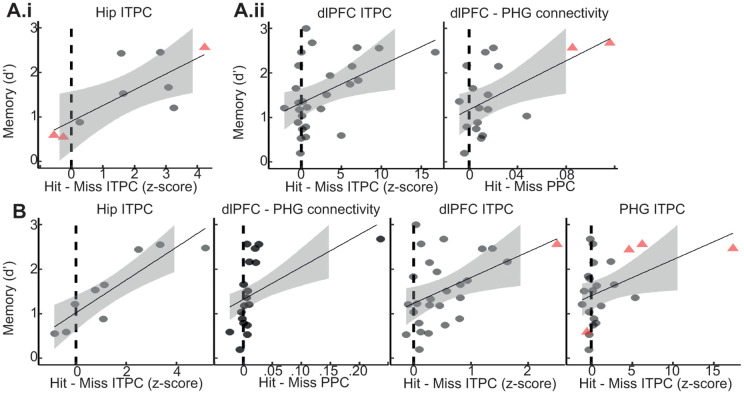
Individual memory performance was predicted by a subset of iEEG signal components. All scatter plots display significant relationships between individual-level signal components and memory performance. The *x*-axis displays the difference between the value of the signal component on hit minus miss trials. The y-axis represents behavioral memory performance (d’). Points represent means across channels within each participant. Red triangles indicate participants whose removal would result in the *p* value rising from below .05 to between .05 and .10. Relationships for which removal of any single participant caused the *p* value to rise above .1 are not shown. The signal component displayed along the *x*-axis of each plot is indicated by its title. **A.i** Signal component aligned to image onset during encoding that predicted memory performance **A.ii** Signal components aligned to HFB peaks during encoding that predicted memory performance. This panel can be regenerated using data contained in allSig.csv and code in allSig.Rmd [112]. **B.** Signal components aligned to image onset during retrieval that predicted memory performance. Note that ITPC at image onset, dlPFC ITPC, and dlPFC-PHG connectivity were important at both encoding and retrieval. This panel can be regenerated using data contained in allSig.csv and code in allSig.Rmd lines 376–460 [112].

Lastly, we conducted an exploratory analysis to correlate each of the 120 significant differences between trials classified based on memory outcome with individual memory performance (Fig 6). Specifically, for each participant, the mean mnemonic difference (subsequent hit versus subsequent miss, or hit versus miss) was calculated across channels per ROI for each signal component at encoding or retrieval. These participant-level values were then correlated with individual memory performance (d’). To ensure that results were not driven by a single participant, correlations are only reported here if they were significant at the p < .05 level and maintained at p < .10 with the removal of any one participant. This exploratory analysis highlights findings that warrant further attention and interpretation but should be interpreted with caution, as our sample sizes were often not large enough for robust analyses of individual differences. During both encoding and retrieval, phase clustering in the Hip and dlPFC (see Fig 3A and 3B) and connectivity between the dlPFC and PHG (see Fig 4E and 4F) predicted individual memory performance. Intriguingly, whereas phase clustering in the dlPFC and connectivity between dlPFC-PHG that predicted memory performance were locked to the HFB peak of the dlPFC during encoding, they were locked to image onset during retrieval. This further emphasizes the late, internally-driven role of the dlPFC during encoding versus its earlier role during retrieval.

Together, these findings reveal that successful memory is supported by transient, selectively timed patterns of connectivity in which temporal organization around both external and internal events is critical.

## Discussion

**Fig 7:**
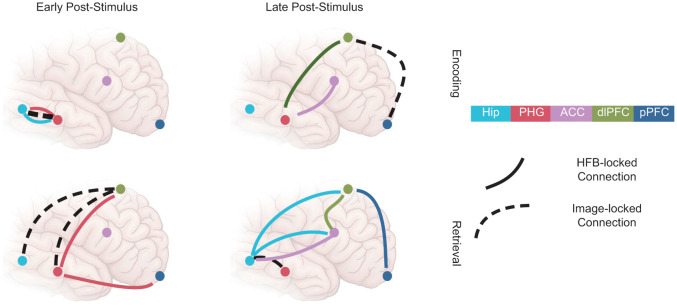
Schematic representation of key findings. Across HFB-locked and image-locked analyses, interregional connections were sparse and transient, demonstrating a cascade of dynamic network states during the successful encoding and retrieval of visual scenes. Of note, during encoding, connections between the PFC and MTL were locked to the timing of HFB peaks in prefrontal regions. During encoding and retrieval, the ACC exhibited connections in HFB-locked analyses only, suggesting that the ACC plays a purely internally-driven role in mnemonic processing.

The present results reveal different sequences of transient states associated with successful memory during encoding versus retrieval across a distributed network of MTL and PFC regions (Fig 7). First, during encoding, PFC-MTL connectivity was absent in the image-locked analysis but present in the HFB-locked analysis, where it predicted individual memory performance. During retrieval, this same connection was aligned to stimulus onset, suggesting a shift from top-down, internally-driven to bottom-up, externally-driven coordination. Second, connectivity involving the ACC was only evident in the HFB-locked analysis, implying that the ACC contributes to internally coordinated processing rather than stimulus-driven responses. Third, graph-theoretic analysis revealed that, regardless of time alignment, transient increases in connectivity between ROIs with memory success were accompanied by a broad reduction in other connections across the brain—a result that challenges prior models based on long-duration connectivity analyses [46,47]. Together, these results emphasize the importance of transient internal events as temporal anchors in large-scale brain dynamics that are at least as important as external events.

We first replicated previous reports indicating that during memory encoding, HFB power peaks in MTL regions prior to PFC regions [9,10,12,13,49], and that there is earlier PFC involvement during successful compared to failed retrieval [7] (see Fig 2D). To better understand the sequence of brain states that accompanied these HFB peaks, all other analyses were performed twice: with time aligned to HFB peak latencies and with time aligned to image onset. This analysis was designed to address a seeming paradox in prior literature. Specifically, if theta connectivity is a primary signal of network organization during cognitive task performance, then why does it seem not to coincide with local processing reflected in HFB activity?

A key idea of our dual approach was that many aspects of brain activity are internally organized rather than directly responsive to external stimulus events [21,50]. Indeed, many effects were not detectable using a typical outside-in approach. This is not to say that analyzing data relative to internal brain events is superior. Five of the seven robust correlations we identified between individual physiology and memory performance were found using metrics aligned to image onset (see Fig 6). Rather, the present results emphasize that the use of internal timing markers augments traditional analysis approaches to yield a fuller picture—particularly for events more distant from stimulus processing.

### Evidence for a transient view of neural dynamics

Three specific findings are worth emphasizing in our results. First, the role of the PFC was different during encoding versus retrieval. In both cases, the PFC exhibited stronger phase clustering in HFB-locked as opposed to image-locked analysis, suggesting attentional orientation to internal information during mnemonic processing in the PFC. These findings extend the observation that low-frequency phase clustering facilitates subsequent behavior through attentional orientation in declarative memory and other cognitive tasks [51–55]. Critically though, the focus of that internal attention appears to have been different. While connectivity between PFC and MTL regions occurred locked to the late HFB peaks of the PFC during encoding, connectivity between these regions occurred locked to the image onset and the early HFB peaks of the MTL during retrieval. These findings suggest that during encoding, the PFC played an internally driven, top-down role, directing activity in the MTL, but during retrieval, the PFC played a more externally driven, bottom-up role, receiving information from the MTL. Critically, the long-distance connections observed during encoding would not have been detectable without HFB-locked analysis (see Fig 7).

Second, within the PFC, connections involving the ACC were only evident in HFB-locked analyses (see Fig 7). This result fits with rodent findings indicating this region as an important node in mnemonic processing [56–58], and supports the idea that the ACC plays a role in conflict resolution and evaluation, which is not directly tied to external processing. Again, this pattern of results would not have been detectable without HFB-locked analysis.

Third, timing is extremely important. Whether considering internally driven HFB-locked connectivity or externally driven image-locked connectivity, our graph-theoretic analysis demonstrated that patterns of connectivity important for both memory encoding and recognition are transient and sparse (see Fig 7). Specifically, we found that at the same time-frequency moments when connectivity was elevated between ROIs, it decreased across the brain. This sparsity is opposite from the broad connectivity increase that has previously been associated with successful memory in prior studies using longer analysis windows [46,47]. These results suggest that successful mnemonic processing at both encoding and retrieval is supported by a series of short-lived, sparsely connected network states. Similarly, it has been shown that decreased between-network communication is associated with better behavioral performance [59]. This may indicate the ability to handle simple tasks, like the one studied here, with a modular (as opposed to integrated) network architecture [60]. More generally, the specific and sparse nature of connectivity revealed by this analysis emphasizes the importance of highly transient events. While we focused here on HFB peaks, the possibility arises that other transient events may be of interest.

### Cognitive processes associated with transient states

The three results highlighted above serve to emphasize the general concepts of internally driven events shaping dynamic network states during complex cognition. However, it is also of interest to integrate the catalogue of 120 significant differences revealed across our analyses to speculate about episodic memory more specifically.

Mean HFB peak latencies occurred early after stimulus onset in both MTL regions. At this time, successful encoding was characterized by theta phase clustering in both regions and theta connectivity between these regions that was locked to their HFB peaks. These physiological effects may represent the transformation of perceptual information into an enduring engram, a process for which the integrity of the MTL is critical and which may be indexed by HFB activity in these regions [29,61]. These mnemonic changes in the temporal organization of theta phase included the prediction of individual memory performance by Hip phase clustering (see Fig 6). Thus, the picture emerges of a Hip-led MTL theta network. Indeed, we and others [16,17,62–64] have also observed Hip theta power increases prior to stimulus onset during successful encoding (see S3 Fig), emphasizing the early involvement of this structure.

Mean HFB peak latencies occurred late after stimulus onset in all regions of the PFC with the pPFC exhibiting the latest HFB peak. Locked to these HFB peaks, there were several signal components that increased during successful encoding. These included theta phase clustering across all three regions, and connectivity between the dlPFC and PHG and between the ACC and PHG for subsequently remembered images. Taken together, these physiological effects may represent inhibition of the PHG by the PFC in order to protect the Hip from interference. Indeed, the PHG is known to act as an informational gate to the Hip, and the anatomical connectivity of the PFC to the PHG could facilitate its control over such a gating mechanism [65–69]. What’s more, at the time of the dlPFC’s HFB peak, low-frequency phase clustering and connectivity with the PHG both predicted individual memory performance (Fig 6), emphasizing the importance of these late effects for memory formation.

During successful retrieval, PFC regions were active earlier in the trial, as has been reported previously [7], and connectivity was increased relative to encoding, as has been reported in rodents [70]. Prior to image onset, successful retrieval was characterized by increased HFB and theta power in the dlPFC (S1 and S3 Figs). These mnemonic effects are not markedly different from those observed prior to image onset during encoding. These effects may index more domain-general processes like attentional or perceptual readiness rather than mnemonic processing itself [33].

Immediately after image onset, hit trials were characterized by a complex flurry of differences from miss trials that contrasted sharply with the equivalent period during encoding when effects had been limited to the MTL. Increased connectivity was observed between the dlPFC and both MTL ROIs between −50ms and 200ms relative to image onset and concentrated between 2 and 3.5 Hz (see S8 Fig). This time period and frequency range overlapped with retrieval-associated phase clustering in both MTL ROIs and the dlPFC, and these effects predicted individual memory performance (see Fig 6; although dlPFC-Hip connectivity only yielded marginal significance *r* = .74; *p* = .06; *n* = 7). Thus, whereas successful encoding was initiated by Hip theta signals, successful retrieval involved a more integrated MTL-PFC network.

Later, after image onset, successful retrieval was characterized by near-simultaneous HFB peak latencies in the Hip, ACC, dlPFC, and pPFC, which was in contrast to the temporal separation of these events observed during encoding. There were other notable differences in this time period from encoding. Whereas MTL-PFC connectivity during successful encoding was observed locked to the HFB peaks of the PFC regions, during successful retrieval, these connections were locked to the MTL’s HFB peaks. It may be that these connections underlie the active processing of memory retrieval itself and the transfer of this information from the Hip to the PFC. However, these connections did not correlate with individual memory performance (*p*s > .5). It could be that late effects reflect recollection and earlier effects reflect familiarity. It is known that the Hip participates in both of these processes [16,71,72], but that they have different behavioral and event-related potential time courses [73,74]. Specifically, familiarity is a feeling of knowing that a stimulus is old (e.g., recognizing acquaintance from stranger), and recollection is the additional retrieval of source information (e.g., hearing the acquaintance’s name) [74,75]. Our recognition memory task did not require recollection per se, which may explain why effects observed during this stage of processing did not correlate with individual memory performance.

### Relationship to ripples

Aligning analyses to HFB peaks is not dissimilar from the growing literature examining the role of ripples in cognitive processing [27,76–78]. Importantly, the term ripple should not be conflated with hippocampal sharp-wave ripple (SWR) complexes. Although averaging individual HFB peak events analyzed here yields grand average waveforms that appear similar to SWR events (S11 Fig), it is not clear what similarities and differences may exist in the source of the signals analyzed here and in SWRs. Recent work on more broadly defined ripple-frequency events similar to the HFB peaks analyzed here has demonstrated that HFB events can co-occur across multiple cortical areas at once, providing a possible mechanism for information integration [79–81]. Our work extends our understanding of these high-frequency events by showing how low-frequency coordination is impacted in sync with these high-frequency modulations. More work is needed to understand the functional roles of different transient HFB events and how they integrate with other signal components. Future work should assess the circuit mechanisms involved in producing different species of HFB events and how they interact with other aspects of network dynamics.

### Limitations and future directions

The present results should be interpreted with caution. First, it is likely that aspects of the brain dynamics reported here develop with age, or may vary with sex or other demographic characteristics. We chose to focus on an adolescent and early adult sample to balance maximization of sample size with minimization of developmental effects, yielding a tighter age distribution than many iEEG studies with similar n. Future research with larger sample sizes should examine how the mnemonic effects reported here may vary across the life span or as a function of sex or other demographic characteristics [82–84]. Second, we chose to focus on a set of brain regions that are known to be involved in memory processing. However, other regions certainly participate as well, and future research should examine how the brain regions examined here interact with others. Third, we did not analyze phase-amplitude coupling (PAC). Given the focus on discrete dynamics, we believe that focusing on phase clustering at particular time points of high HFB power captures the relationship between HFB and theta signaling effectively. PAC analyses either require long temporal windows encompassing multiple cycles of the low-frequency signal or, if done at the singular time point of HFB peak across trials would be equivalent to HFB-locked phase clustering presented here. Fourth, it is known that eye movements are associated with modulation of gamma activity during memory formation [85]. Although eye movements were not recorded in the present study, it would be of interest to examine how our results may be modulated by eye movements. Fifth, we defined a singular HFB peak per trial as the time point of maximum HFB power. Although this afforded us the greatest opportunity to study strong and meaningful physiological events, secondary peaks within trials may have represented physiologically meaningful events as well, and future research should work to establish criteria for event detection and categorization. Finally, with any iEEG study there is a balance between anatomical specificity and sample size. In our analysis we focused on relatively large ROIs, and our analysis was agnostic to hemisphere. Anatomical variability at finer scales and between hemispheres certainly exists and likely introduced extra variability into our analysis. Fine anatomical differences would have introduced variance and decreased our chances of observing statistical significance. Future studies will likely reveal further nuance with sample sizes sufficient to consider finer anatomical distinctions.

### Conclusions

We identified a sequence of transient processing states that support recognition memory, characterized by coordinated changes in theta phase clustering, low-frequency connectivity, and HFB activity. These mnemonic states were sparsely distributed in time and space, and their detection depended critically on aligning analyses to both internal (HFB peaks) and external (stimulus onset) temporal anchors. Critically, many of our findings could not have been revealed using traditional stimulus-aligned analyses. Thus, our results emphasize the importance of internally-driven brain events as temporal anchors for complex network dynamics. This work reframes how local activity and global coordination jointly support memory through brief windows of targeted communication embedded within ongoing neural dynamics.

## Star methods

### Experimental model and study participant details

Participants were 36 adolescents and adults (22 males; 13–28 years of age; M ± SD, 19.0 ± 4.7 years) undergoing intracranial monitoring as part of clinical management of seizures. We did not have access to participants’ ancestry, race, ethnicity, or socioeconomic status. Demographic and behavioral data are provided in S1 Table. Subjects were selected from a larger pool based on non-pathologic sampling of regions of interest (ROIs; i.e., electrodes localized to the Hip, PHG, ACC, dlPFC, and/or pPFC and outside seizure onset zones [86]), age greater than 13 years, and memory accuracy above chance (see below). There is partial overlap in subjects between this study and earlier studies using the same memory task [31–35]. Subjects were recruited from Northwestern Memorial Hospital (IRB #STU00215843), the Ann & Robert H. Lurie Children’s Hospital of Chicago (IRB #2022-5020), the Children’s Hospital of Michigan (IRB #048404MP2E), the University of California, San Diego Rady Children’s Hospital and the University of California, Irvine Medical Center (IRB #2014-1522), the University of California, Davis Medical Center (IRB #1623773-1), Nationwide Children’s Hospital (IRB #2020N0022), California Pacific Medical Center (IRB #666687-17), and St. Louis Children’s Hospital (IRB #201102222). Written informed consent was obtained from subjects aged 18 years and older and from the guardians of all subjects younger than 18 years; written assent was obtained from subjects aged 13–17 years. All consent procedures were performed in accordance with the Declaration of Helsinki.

### Method details

#### Behavioral task.

Subjects performed a scene recognition memory task (Fig 1A) that has been used extensively to delineate the functional architecture of memory development with fMRI and iEEG [31–35,82,87–91]. Subjects studied sets of 40 indoor and outdoor scenes, each shown for 3 s following a 0.5-s fixation interval. Stimuli were full-color pictures of natural scenes, half of high complexity and half of low complexity, characterized based on the number of object categories (over/under four) depicted [32,92]. During the encoding phase, subjects were instructed to indicate verbally whether each studied item depicted an indoor or an outdoor scene. Responses were coded as correct or incorrect via offline review of individual audio recordings. A fixation cross remained on screen until a response was provided, if none was provided during the 3-s scene presentation epoch. Per-trial RTs were automatically calculated by subtracting scene onset times from verbal response onset times. Analysis of electrophysiological data was restricted to trials in which scenes were correctly classified as indoor/outdoor, indicating the scenes were properly attended during the study block [31–35]. In addition, only trials with RTs below 3 seconds were considered.

The memory recognition test included all 40 scenes presented during the encoding block, intermixed in a randomized order with 20 new scenes. Following a 0.5-s fixation pretrial interval, each scene remained on screen until a response was given. Subjects verbalized an old/new judgment for each scene, which was coded as a hit (correct old), miss (new response to an old scene), correct rejection (CR; correct new), or false alarm (FA; old response to a new scene) via offline review of individual audio recordings. RTs were automatically calculated by subtracting scene onset times from verbal response onset times, and trials were excluded if no response was given.

This procedure was repeated twice, yielding total trial counts of 80 encoding trials, 80 retrieval trials with old images, and 40 retrieval trials with new images. All subjects completed a short practice run and at least one full encoding-test run. Recognition accuracy was calculated as hit rate minus false alarm rate [31–35]. Only subjects with recognition accuracy greater than 0 were included in the present analysis.

The present analysis focuses on subsequent hit, subsequent miss, hit, and miss trials.

#### Electrode placement and localization.

Macro-electrodes were surgically implanted for extra-operative recording based solely on the clinical needs of each patient. Electrodes were placed subdurally in 4- to 8-channel strips and 2–8 by 8 channel grids with 10-mm spacing (i.e., ECoG) or stereotactically in 8- to 18-channel tracks with 5- or 10-mm spacing (i.e., sEEG). Anatomical locations were determined by co-registering post-implantation computed tomography (CT) coordinates to pre-operative magnetic resonance (MR) images, as implemented in FieldTrip [93]. Electrodes were localized in native space based on visual inspection of individual anatomy. For group-level visualization, electrode locations were transformed into standard MNI space.

#### Data acquisition and preprocessing.

EEG data were acquired using Nihon Kohden systems, sampled between 0.5 and 5 kHz, and Natus systems, sampled between 0.512 and 2.048 kHz. The BCI2000 software was used to acquire and store data in a subset of subjects. Raw EEG data were filtered with 0.1-Hz high-pass and 300-Hz low-pass finite impulse response filters, and 60-Hz line noise harmonics were removed using discrete Fourier transform. Continuous data were demeaned, epoched into 4.5-s trials (−1 to +3.5 s from scene onset), and manually inspected blind to electrode locations and experimental task parameters. Electrodes overlying seizure onset zones [86] and electrodes and epochs displaying epileptiform activity or artifactual signal (from poor contact, machine noise, etc.) were excluded, ensuring that the data analyzed would represent healthy tissue [94]. Neighboring artifact-free electrodes within the same anatomical structure were then bipolar referenced using consistent conventions (ECoG: anterior–posterior, sEEG: deep–surface) to form virtual channels anatomically located halfway between the two contributing electrodes [40,95,96]. An electrode was discarded if it did not have an adjacent neighbor, its neighbor was in a different anatomical structure, or both it and its neighbor were in white matter. Bipolar referencing was used to minimize contamination from volume conduction [97]. Data were then manually re-inspected to reject any trials with remaining noise. Functions from the FieldTrip toolbox for MATLAB were used for preprocessing routines [98].

#### Electrode selection by regions of interest.

Bipolar channels were selected for analysis in the present study if they fell into one of five ROIs: pPFC, dlPFC, ACC, PHG, or Hip. The pPFC was defined as Broadman’s area 10. The dlPFC was defined as the posterior two-thirds of the superior and lateral surface areas of the superior frontal gyrus, the posterior two-thirds of the middle frontal gyrus. And the posterior one-third of the inferior frontal gyrus. The ACC was defined as Broadman’s areas 32, 33, and 25, as well as the anterior half of area 24. The PHG was defined as the combined perirhinal, entorhinal, and parahippocampal cortices [99]. The Hip was defined as the entire volume of the dentate gyrus, fields of cornu ammonis, and subiculum. Across subjects, there were 35 channels in the pPFC, 247 in the dlPFC, 44 in the ACC, 79 in the PHG, and 40 in the Hip.

### Quantification and statistical analysis

#### Signal processing analysis approach.

Although the analysis presented here focuses on five ROIs, key signal components were extracted for all recorded channels. This approach facilitated graph theoretical analysis (described below), but was computationally intensive, requiring parallelization of analysis code for computation on a high-power cluster (HPC). Coding principles to facilitate this analysis approach have been described previously [100]. Briefly, each participant’s data were divided into a set of individual channel files. Signal processing was then performed on these individual channel files in parallel, and inferential statistics were performed on ROI groups of channel files after signal processing was complete. Key signal processing steps described in the remainder of this sub-section were all executed in the function singleChanPipeline. All signal components were down-sampled after extraction to a sampling rate of 40 Hz to reduce file sizes and computation time.

HFB time series were extracted. Data were narrowband filtered using the Fieldtrip [98] function ft_specest_mtmconvol between 70 and 150 Hz using ‘dpss’ (i.e., multitaper analysis [101]) for the taper argument and padding equal to the next power of two divided by the sample rate. Filtered data were converted into power time series by absolute valuing and squaring. The mean was taken across tapers. Power time series were z-scored within frequency based on the mean and standard deviation of a pre-stimulus baseline period of −450 to −50 ms relative to image onset using a bootstrap procedure implemented in myChanZscore and described previously [32,33,38,40,95,96]. Finally, a single HFB time series was obtained by taking the mean across frequency of the z-scored power time series. See functions getHFB, myChanZscore, and getChanMultiTF for more details.

Only channels whose HFB time series exhibited evidence of task reactivity were included in inferential statistics and plots. Each channel was deemed reactive if its mean HFB time series across trials exceeded *z* = 1.96 for any continuous 50 ms period between −50 ms and 2,000 ms relative to image onset or −1,500 ms and 500 ms relative to the behavioral response [102]. Reactivity was assessed at both encoding and retrieval. Channels that were reactive for any one period were used in all analyses. Reactivity was calculated with data aggregated across all trial types. See functions checkForThreshold_100 and reactiveTest_100 for details.

The latency of peak HFB activity was calculated for each trial. Each trial was smoothed with a 275-ms Gaussian kernel convolved with the HFB time series. The latency of the peak of the smoothed data between image onset and the behavioral response was extracted. See function gausLat for details.

Morlet Wavelet convolution in the frequency domain was used to extract narrowband complex time series at 100 logarithmically spaced frequencies between 2 and 80 with corresponding standard deviations ranging from 3 to 10 [103]. For each frequency, the resulting analytic signal was converted into a phase time series using the MATLAB function angle and a power time series using absolute value and squaring. Power time series were z-scored within frequency in the same way as HFB time series. See functions getChanTrialTF and myChanZscore for details. Inter-trial phase clustering (ITPC) was calculated using the phase time series at the time of inferential statistics [103].

Pairwise phase consistency (PPC) [45] was extracted between all pairs of channels for all trial types across 20 logarithmically spaced frequencies between 2 and 25 Hz. These connectivity metrics were calculated across trials for each time point, with trials aligned in time to the onset of the image. In addition, these metrics were also calculated across trials for each time point with trials aligned to the HFB peak latency. Because HFB latency was different for different channels, the HFB latency-aligned connectivity analysis was non-symmetric between regions. For example, connectivity was calculated between the Hip and PHG using time aligned to the peak of the HFB in the Hip, and it was also calculated using time aligned to the peak of the HFB in the PHG. Calculation of PPC was done using custom code. Different trial types were analyzed separately. See function getChanISPC for details.

#### HFB-locked and image-locked temporal alignment.

Inferential statistics were done in two ways for all signal components. HFB-locked analyses used the latency of the HFB peak in each trial as *t* = 0, and data points surrounding the HFB peak were extracted for analysis + /-500ms around this time point for each trial. Image-locked analyses used the latency of the image onset on the computer screen as *t* = 0 and data points surrounding the image onset were extracted for analysis −450–3,000 ms for each trial.

#### Within-ROI inferential statistics.

Within-ROI hit/miss differences were examined independently for each ROI and for encoding/retrieval using a standardized method across all signal components and both HFB-locked and image-locked temporal alignments. Specifically, this method was used for HFB power, low-frequency power, low-frequency phase reset (ITPC), and PPC data. Inferential analysis focused on measuring differences between (subsequent) hit versus miss trials. Linear mixed-effects (LME) models were applied independently to model different signal components and for encoding and retrieval data. For all analyses hit/miss was a fixed effect and channel and subject ID were random effects [104,105].

Because signal components varied across both time and frequency, LME models were applied at every time and frequency point independently. To correct for multiple comparisons, cluster-based permutation testing was applied [106,107]. Specifically, the channel mean for (subsequent) hit and miss trials was calculated for each measure at each time-frequency point. Next, the hit and miss identity of each channel’s observed values was shuffled within channel and the model was refit. This process was repeated 2,500 times. The function cluster_test was used to evaluate significance of the observed model output versus the permutation outputs [106]. Specific functions to generate the permutations and fit the LME models were written for each signal component separately: HFBsingleTrialPipeline.m, connectivityPipeline.m, TFphaseTrialpipeline.m, TFsingleTrialpipeline.m.

#### HFB latency between ROI inferential statistics.

HFB latency values for all individual trials along with subject ID, channel ID, encoding/retrieval, hit/miss, and behavioral RT were aggregated and exported from MATLAB for modeling in R using the Tidyverse for general data handling [108], and the lme4 package for modeling [109]. HFB latency was modeled as a function of the fixed effects of hit/miss * encoding/retrieval * region with subject ID and channel ID used as random effects (Fig 2D). Holm-corrected pairwise contrasts were evaluated to aid interpretation of significant effects in the model using the R function emmeans [110].

A major challenge with this analysis was that different participants contributed channels from different ROIs. Thus, it was difficult to know if differences in the relative timing of HFB latency between ROIs were due to differences in regional brain function or differences between individual subjects. To mitigate this confound, this analysis was carried out in 3 ways. First, a bias-corrected measure of HFB latency was modeled (main text). Specifically, HFB latency was divided by RT to provide a measure that ranged from 0 to 1 and accounted for individual differences in RT. Second, the raw HFB latency was modeled (S2A Fig). Third, a subset of trials with matched RTs was selected and these were used for modeling (S2B Fig). Fourth, in the matched subset of trials HFB latency was divided by RT and these bias-corrected values of the matched trials were used for modeling (S2C Fig). The Anova function in R was used to extract chi-squared and p values from these models using Type II Wald tests. The emmeans function with holm adjustment for multiple comparisons was used to perform pairwise contrasts between regions within different trial types and also between different trial types within region.

As an additional check, for pairwise sets of ROIs, the single-trial HFB latencies were compared in simultaneously recorded pairs of channels (S2D Fig). Histograms were plotted of the relative HFB latency for these within-subject comparisons. In addition, ROI label was shuffled to generate a null distribution. The difference between these distributions was evaluated with KS-test. A key question was whether the directionality of these within-subject comparisons would corroborate the results of the omnibus between-subject analyses described in the previous paragraph. All analyses in this section were executed in Latency_LME_modeling.Rmd.

#### HFB-locked power spectrum between ROI inferential statistics.

Visual inspection of time-frequency plots centered on HFB peaks indicated that power increases were highly concentrated at the time point of the peak itself (S3A and S3B Fig), and so instantaneous estimates of the power spectra at this time point were examined alone (S3C Fig). Differences in the power spectra observed were explored using the lmer function in R. Power was modeled as a function of frequency (2–80 Hz; modeled with 8 splines) * region * encoding/retrieval with subject ID and channel ID used as random effects. The ANOVA function in R was used to extract chi-squared and p values from these models using Type II Wald tests. These analyses are implemented in GAM_analysis_HFB_spectra.Rmd.

Because frequency was a continuous variable and was modeled non-linearly, it was not straightforward to extract post-hoc contrasts to explain significant effects in the full model. Instead, we took a descriptive approach. We reasoned that if peak frequencies in the power spectra varied between ROIs and conditions, then these differences in power likely contributed to the effects in the full model. To do this, a low spectral peak was identified for each ROI during encoding and retrieval by identifying the frequency at which power was maximal between 2 and 20 Hz. A medium spectral peak was identified by searching for a second peak above the low peak and below 34 Hz. The presence of a medium spectral peak was defined to exist when the maximum power value higher than the first trough after the low peak and below 34 Hz was a higher value than the power at either of the endpoints of this range. The key idea is that any differences in the shape of the power spectra will drive differences in the omnibus model. Thus, the particular choice of descriptive methodology does not influence whether or not power values varied as a function of frequency, region, or encoding/retrieval. The point is simply to describe how the shape of the power spectrum changed across conditions.

#### Contrasting ITPC between image and HFB locked analyses (IMG-HFB index).

An IMG-HFB index was used to quantify the relative strength of ITPC differences in HFB-locked versus image-locked analyses (Fig 3C–3E). To do this, for each region the frequency of maximal difference in HFB-locked ITPC between hit and miss trials was chosen for examination. The Raleigh’s Z magnitude of ITPC at this target frequency in HFB-locked data for hit and miss trials was extracted for each channel. A new frequency was chosen similarly for image-locked data. This resulted in one ITPC value for each of four conditions for every channel: HFB-locked hits, HFB-locked misses, image-locked hits, and image-locked misses. Corresponding HFB and image-locked values were used to create an index score for each channel and trial type reflecting the relative strength of ITPC values across the two time alignment methods:


$${IMG-HFBindex=(ITPC}_{HFB}-{ITPC}_{image})/({ITPC}_{HFB}+{ITPC}_{image})$$

This index could take on values between −1 and +1 where −1 indicated that image-locked ITPC was much stronger than HFB-locked ITPC. It turned out that for 40% of trial types, both time-alignments had the exact same frequency of maximal ITPC difference between hit and miss trials. For 84% of trial types, the two time-alignments yielded frequencies of maximal ITPC difference that were within 2 Hz of each other.

IMG-HFB index values were modeled using linear mixed effects modeling as a function of region, hit/miss, and encoding/retrieval. This analysis followed the same approach as the latency analysis described above and was executed in HFB_IMG_index.Rmd.

#### Visualization of connectivity results.

To aid interpretation, ROIs were plotted schematically as dots, and connections that significantly increased in strength during hit trials over miss trials were plotted as lines connecting ROIs (Fig 4E and 4F). Heatmaps displaying granular time-frequency plots of PPC for individual connections are available in S6–S9 Figs; see S5 Fig for equivalent figures displaying significant negative connectivity differences. These schematic connectivity plots were generated for HFB-locked (Fig 4E) and image-locked (Fig 4F) analyses separately, and connections were further grouped by time periods and encoding versus retrieval.

#### Graph theoretic analysis.

Every significant difference between two ROIs in PPC between hit and miss trials was termed a mnemonic connection. The mean time and frequency of the significant difference was extracted. Using this time and frequency point, the full channel X channel connectivity matrix was extracted for every participant with channels in both ROIs of the mnemonic connection under consideration. These full channel X channel matrices included many channels outside ROIs, but the goal of this analysis was to understand the whole-brain network state at the moments of mnemonic connections. Channel X channel matrices were extracted for hit and miss trials independently. Mnemonic connections were assessed for encoding and retrieval separately.

Next, three graph theoretic measures were calculated for each channel X channel matrix using functions from the Brain Connectivity Toolbox [48]. Weighted graph analysis treats each channel as a node and PPC values as the strength of the connecting edges of the network. Unweighted graph analysis is similar but requires that connections be binarized into values of either 0, indicating no connection along a possible edge, or 1, indicating a connection along a possible edge. When necessary, channel X channel connectivity matrices were binarized with a threshold of PPC > .1 =< 1; PPC < .1 => 0, but the choice of threshold did not qualitatively affect results.

Analysis focused on three measures. The weighted characteristic path length was calculated using the BCT functions charpath and distance_wei and described the average distance of paths between any two nodes in the network. The weighted strength was calculated using the BCT function strengths_und and was simply the sum of PPC values observed for each channel. The unweighted strength was calculated as the average proportion of edges with PPC > .1 for each channel. Strength values were averaged over channels to obtain a single estimate for the full network.

Although channel coverage varied over participants, it is important to note that our main findings from this analysis focused on hit/miss differences rather than on the absolute value of graph measures (Fig 5). The LME approach models independent random intercepts for each participant, which accounts for the biasing difference of channel coverage, allowing for valid inferences about fixed effects of hit/miss. By contrast, regional differences in these measures must be interpreted with caution as different participants contributed observations from different regions. As such, regional differences are presented in the supplement only (S10 Fig).

LME modeling of graph measures followed a similar approach as presented above for HFB latency and each graph measure was modeled as a function of region * hit/miss * encode/retrieve in graphMeasures.Rmd.

These were weighted characteristic path length, unweighted strength, and weighted strength. Weighted characteristic path length uses the PPC values as edge strengths signifying.

These channel X channel connectivity matrices were extracted and graph measures combined for statistical analysis in connectionGraph.m and finalGraphDatCombine.m. LME modeling was done in graphMeasures.Rmd.

#### Correlations with memory performance.

For every significant difference between hit and miss trials across all signal components, individual memory performance (measured using *d*’ [111]) was compared with individual differences between hit and miss trials. To do this, the significant cluster of time and frequency points for each hit miss difference was used as a mask to select the relevant time-frequency extent of the difference. For each participant, this mask was used to select data points from the mean hit signal and from the mean miss signal. Next, a difference score between hit and miss trials was calculated across the selected data points and the mean difference was calculated. When a participant had more than one channel that contributed to a significant difference, the mean hit/miss difference was calculated across channels for that participant. In this way, each participant contributed a single memory performance value and a single hit/miss signal difference value. Because of differences in channel coverage, the n associated with each signal component and regional difference varied. These values were extracted using the script publicationFigures.m and exported for analysis in R using the script allSig.Rmd.

Next, individual hit/miss signal differences and memory performance values were correlated with each other. For all significant correlations (*p* < .05), a leave-one-out procedure was performed. For this procedure, each participant was left out one at a time, and the correlation was recalculated. Only those correlations that were significant in the full group at the 0.05 level and that maintained significance at the 0.10 level after removal of any one participant are reported in Fig 6. Given that n was often small and that correlation values can vary dramatically with single data points when n is small, this procedure ensured that none of the reported correlations were reliant on a single participant.

This analysis was exploratory and its goal was not to establish causal relationships between physiological measures and memory performance. Rather, the goal of this analysis was to aid interpretation by providing an additional statistical check beyond the rigorous LME and cluster permutation testing to which each hit/miss difference had already been subjected. In this way, from the large number of significant effects discovered and reported here, it was possible to highlight significant differences of particular promise for interpretation and future investigation.

## Supporting information

S1 FigHFB power changes aligned to image onset and HFB peak.**A**. Each line plot displays the mean time series of the HFB response across channels within different regions. For all panels, the x-axis displays time relative to image onset, and the y-axis displays HFB power in z-scored units. Encoding and retrieval data are plotted along the top and bottom rows, respectively. Orange and blue lines are the average time series of (subsequent) hit and miss trials, respectively. Vertical gray shaded regions indicate *p* < .05 for the difference between hit and miss after cluster correction. Successful encoding was associated with elevated HFB activity prior to image presentation in the dlPFC, and ~500 ms after image presentation and after the indoor/outdoor behavioral response in the pPFC. Successful retrieval was associated with elevated HFB activity prior to image presentation in the dlPFC, but lower HFB activity for failed memory late in the trial in both the dlPFC and pPFC. The PHG exhibited effects during both encoding and retrieval, but these were much later and smaller in magnitude than the visual response. Colored shaded regions indicate the standard error of the mean. Colored vertical dashed lines indicate mean reaction times for hit trials (blue) and miss trials (orange). This panel can be regenerated using data contained in the HFB_image folder and code in SupFigure1A.m [112]. **B**. Each grouped scatter plot displays the mean power of HFB peak for each channel grouped by region. Mean z-scored HFB power at the time of the HFB peak is displayed on the *y*-axis. Error bars display the 83% confidence interval around model estimates [113,114]. Linear mixed effects modeling of these data revealed main effects of encode/retrieve $\left(\chi^{\text{2}}(\text{1})=\text{1}\text{8}\text{8},p<\text{2}e-\text{1}\text{6}\right)$ and hit/miss $\left(\chi^{\text{2}}(\text{1})=\text{1}\text{1},p=.\text{0}\text{0}\text{0}\text{8}\right)$, and all four interaction terms: hit/miss by encode/retrieve $\left(\chi^{\text{2}}(\text{1})=\text{6},=.\text{0}\text{1}\text{6}\right)$, hit/miss by region $\left(\chi^{\text{2}}(\text{1})=\text{1}\text{1},p=.\text{0}\text{2}\right)$, encode/retrieve by region $\left(\chi^{\text{2}}(\text{1})=\text{1}\text{4},p=.\text{0}\text{0}\text{7}\right)$, and hit/miss by encode/retrieve by region ${(\chi }^{\text{2}}(\text{4})=\text{2}\text{0},p=.\text{0}\text{0}\text{0}\text{5})$. Holm-corrected pairwise contrasts revealed that in the PHG, Hip, dlPFC, and pPFC, the peak HFB power was higher during retrieval than encoding. Mnemonic effects were evident in the ACC and PHG, with higher peak power during failed encoding than during successful encoding. This panel can be regenerated using data contained in trialLatDat_RTfix.csv and code in Latency_LME_modeling.Rmd lines 957–1,069 [112]. **C**. Mean HFB power time series using the same conventions as in A except with time centered around the latency of the HFB peak. Both panels display data from the encoding phase of the experiment, with ACC data displayed on top and PHG data below. Note that although the image-locked mean HFB activity in the ACC appeared flat (panel A), its peak activity was no smaller than in any other region. This panel can be regenerated using data contained in the HFB_hfb folder and code in SupFigure1C.m [112].(PDF)

S2 Fig Timing of HFB peak latency can be measured in several ways, but all reinforce the same general interpretation. **A.** Each grouped scatter plot displays the mean time of peak HFB latency for each channel grouped by region. Time relative to image onset is displayed in milliseconds on the *y*-axis. Error bars display the 83% confidence interval around model estimates [113,114]. **B.** Similar to A except a subset of trials was selected such that reaction time was matched at the individual trial level. **C.** Similar to B, except that a subset of trials was selected, such that reaction time was matched at the individual trial level, and normalized time was used. **D**. The asynchrony of HFB latency within trial is displayed for pairs of channels recorded simultaneously within individual participants. The four groups of figures represent the behavioral conditions indicated by the large font labels in the left and top margins. The *x*-axis shows the difference in timing of the HFB peak observed on single trials at pairs of simultaneously recorded channels. Positive values correspond to the brain region indicated at the top of the column that had an earlier HFB peak latency. Negative values correspond to the brain region indicated at the side of the column that had an earlier HFB peak latency. The *y*-axis shows the proportion of trials observed to have a given peak latency asynchrony. The color of the line represents the brain region with the earlier latency (as judged by proportion of trials). The dashed line shows the latency asynchronies observed when region identity is shuffled prior to calculating latency asynchrony. The percent of trials where the row region was the leader is indicated in the upper left of each plot. The percent of trials where the column region was the leader is indicated in the upper right. Note, these values do not sum to 100% because ties were discounted. The pairwise contrasts observed here recapitulate the order observed in Fig 2D of the main text. All panels can be regenerated using data contained in trialLatDat_RTfix.csv and code in Latency_LME_modeling.Rmd lines 736–824 (A), 833–921 (B), 615–727 (C), and 139–319 (D) [112].
(PDF)

S3 FigTF power differences between hit and miss trials across encoding and retrieval.**A**. heatmaps display frequency in Hertz on the *y* axis and time in milliseconds relative to HFB peak on the *x* axis. Color indicates power z-scored within frequency. The top row reflects data collected during subsequent hit trials. The bottom row reflects data collected during subsequent miss trials. **B**. Similar to A except for retrieval. **C**. To facilitate comparison between hit and miss trials, line plots display power spectra at the time point of the HFB peak. For all panels, the x-axis displays frequency, and the y-axis displays power in z-scored units. Encoding and retrieval data are plotted along the top and bottom rows, respectively. Orange and blue lines are the average spectra of (subsequent) hit and miss trials, respectively. Vertical gray shaded regions indicate p < .05 for the difference between hit and miss spectra after cluster correction. Colored shaded regions indicate the standard error of the mean. Successful encoding elicited greater power in the Hip between 2.0 and 2.9 Hz. Failed retrieval elicited greater power in the PHG between 4.4 and 15 Hz. Although other ROIs did not exhibit hit/miss differences, power spectra nevertheless appeared different across regions and between encoding and retrieval. These differences were explored using linear mixed effects modeling of power as a function of frequency (2–80 Hz; modeled with 8 splines), region, and encoding/retrieval. All interactions between fixed effects were significant $\left(\chi^{\text{2}}(\text{4}-\text{3}\text{2})>\text{6}\text{6},maximump<\text{2}e-\text{1}\text{0}\right)$. Several factors may explain these interactions. First, an interaction between frequency and region may have been driven by regional variations in the frequency of maximum power in the lower range (2–20 Hz; S1 Table). Second, an interaction between encode/retrieve and region may have been driven by higher power in the Hip during retrieval than during encoding. Third, a three-way interaction may have been driven by secondary power increases between 24 and 32 Hz in the Hip, ACC, and dlPFC during retrieval (S1 Table). These results emphasize that HFB peaks constituted physiological events, and not statistically extreme values. These panels can be regenerated using data contained in the TF_HFB folder and code in SupFigure3ABC.m [112]. **D**. Similar to A, except time on the *x*-axis is represented in seconds relative to the image onset. **E**. Similar to D, except for retrieval. **F**. Heatmaps display the mean difference in z-scored power between hit and miss trials. For all panels, the *x*-axis displays time relative to image onset, and the y-axis displays frequency. White outlined areas indicate *p* < .05 for the difference between hit and miss power after cluster correction. In the Hip and dlPFC, there were positive subsequent memory effects in 2–10 Hz power just before and during image onset (−450–100 ms). In the PHG, there was a negative memory effect in 2–10 Hz later after image onset at both encoding (550–1,550 ms) and retrieval (925–1,450 ms). These decreases partially overlapped with the visual response observed in the PHG’s HFB power (S1A Fig). Finally, the pPFC exhibited a late (1,225–2,775 ms) positive subsequent memory effect during encoding across 2–10 Hz. At retrieval, the pPFC exhibited a much earlier (−100–600 ms) positive memory effect and a later (950–1,975 ms) negative memory effect. These panels can be regenerated using data contained in the TF_image folder and code in SupFigure3DEF.m [112].(PDF)

S4 Fig Distributions of phase preference of phase clustering within channels. **A**. Histograms display the proportion of channels on the *y* axis and the phase of either the 3 Hz (left columns) or 6.5 Hz (right columns) component of the signal. For each channel, the mean circular phase was calculated across trials at the time point of the HFB peak for hit (blue) and miss (orange) trials separately. The superimposed sin wave on each plot indicates which phase values correspond to the peak, trough, or intermediate positions in the oscillation. The dashed-dotted lines indicate uniform distribution. The top row of each panel displays encoding data. The bottom row displays retrieval data. Each panel displays data for a different region. Notice that the Hip exhibited a preference for HFB activity to occur at the trough of the 3 Hz oscillation during successful memory trials at both encoding and retrieval, but the PHG exhibited a preference for the peak. This panel can be regenerated using data contained in the TFphase_HFB folder and code in Figure3A_supFigure4A.m [112]. **B**. Similar to A, but mean phase was measured at the time point of maximal ITPC relative to image onset. Notice that phase preferences evident in MTL regions in C appear weaker when measured relative to image onset. This panel can be regenerated using data contained in the TFphase_image folder and code in Figure3B_supFigure4B.m [112].
(PDF)

S5 Fig Negative mnemonic connections. Plots A and B correspond to main text Fig 4E and 4F, respectively. However, while the schematics in Fig 4E and 4F display only connections that exhibited increased strength for hit trials, this figure displays only connections that exhibited decreased connectivity strength for hit trials relative to miss trials. Note that far fewer of these negative connections were detected than the positive connections shown in Fig 4. These panels can be regenerated using data contained in HFBConnections.mat and imageConnections.mat and code in Figure4EF.m [112].
(PDF)

S6 Fig Inter-regional connectivity changes associated with memory during encoding. Analysis aligned to image onset. In all panels, subplots have time relative to image onset (t = 0) on the X-axis and frequency on the Y-axis. The color scale indicates the strength of connectivity between the regions indicated by the row and column of the subplot. **A.** Connectivity during subsequent hit trials using pairwise phase consistency. **B.** Connectivity during subsequent miss trials measpured using pairwise phase consistency. **C.** The difference between hit and miss trials. Warm colors indicate stronger connectivity during hit trials. Color scale represents t-values. White outlines indicate cluster-corrected significant differences between hit and miss trials. These panels can be regenerated using data contained in the connectionDat folder and code in SupFigure6_7_8_9_10A.m [112].
(PDF)

S7 Fig Inter-regional connectivity changes associated with memory during encoding. Analysis aligned to HFB peak of the region indicated by the row position of each subplot. In all panels, subplots have time relative to HFB peak (t = 0) on the X-axis and frequency on the Y-axis. The color scale indicates the strength of connectivity between the regions indicated by the row and column of the subplot. **A.** Connectivity during subsequent hit trials measured using pairwise phase consistency. **B.** Connectivity during subsequent miss trials measured using pairwise phase consistency. **C.** The difference between hit and miss trials. Warm colors indicate stronger connectivity during hit trials. Color scale represents t-values. White outlines indicate cluster-corrected significant differences between hit and miss trials. These panels can be regenerated using data contained in the connectionDat folder and code in SupFigure6_7_8_9_10A.m [112].
(PDF)

S8 Fig Inter-regional connectivity changes associated with memory during retrieval. Analysis aligned to image onset. In all panels, subplots have time relative to image onset (t = 0) on the X-axis and frequency on the Y-axis. The color scale indicates the strength of connectivity between the regions indicated by the row and column of the subplot. **A.** Connectivity during hit trials measured using pairwise phase consistency. **B.** Connectivity during miss trials measured using pairwise phase consistency. **C.** The difference between hit and miss trials. Warm colors indicate stronger connectivity during hit trials. Color scale represents t-values. White outlines indicate cluster-corrected significant differences between hit and miss trials. These panels can be regenerated using data contained in the connectionDat folder and code in SupFigure6_7_8_9_10A.m [112].
(PDF)

S9 Fig Inter-regional connectivity changes associated with memory during retrieval. Analysis aligned to HFB peak of the region indicated by the row position of each subplot. In all panels, subplots have time relative to HFB peak (t = 0) on the X-axis and frequency on the Y-axis. The color scale indicates the strength of connectivity between the regions indicated by the row and column of the subplot. **A.** Connectivity during hit trials measured using pairwise phase consistency. **B.** Connectivity during miss trials measured using pairwise phase consistency. **C.** The difference between hit and miss trials. Warm colors indicate stronger connectivity during hit trials. Color scale represents t-values. White outlines indicate cluster-corrected significant differences between hit and miss trials. These panels can be regenerated using data contained in the connectionDat folder and code in SupFigure6_7_8_9_10A.m [112].
(PDF)

S10 FigAdditional graph analysis results.**A.** Box plot display connectivity between the ACC and Hip for all channel pairs that spanned the two regions (open blue circles) and participants (red circles) averaged across the same time and frequency windows as in Fig 5A.i and 5A.ii. Note that although miss trials were characterized by more densely connected overall graphs (Fig 5A.ii), connectivity between the ACC and Hip was weaker during miss trials for all participants. This panel can be regenerated using data contained in the connectionDat folder and code in SupFigure6_7_8_9_10A.m [112]. **B.** The histogram displays the strength of all connections for the representative participant shown in Fig 5A averaged across the long temporal epoch (corresponding to Fig 5A.iii and 5A.iv). Note that with lower variance in the hit distribution, a higher central tendency in the hit distribution, and similar representation at very strong connectivity strengths between hit and miss trials (see inset), a z-scored analysis would replicate prior findings of stronger connectivity for hit trials [47]. Overall, for this example participant, 52% of all possible connections exhibited greater connectivity values for successful encoding over failed encoding, and the corresponding value was 54% for retrieval trails. It is interesting that overall connectivity was stronger during hit trials when examined over the longer temporal window. This panel can be regenerated using data contained in hip_acc_ret_HFB_2_21.mat and code in Figure5A_supFigure10B.m [112]. **C.** Same as in Fig 5B but with data sorted by whether significant connections were detected in HFB-aligned or image-aligned analyses. There was a modest effect such that graphs exhibited more connectivity for HFB-locked analyses than image-locked: shorter characteristic path length, increased weighted strength, and increased unweighted strength; $\chi^{\text{2}}(\text{1})>\text{1}\text{2},maximump<.\text{0}\text{0}\text{0}\text{5}$. This panel can be regenerated using data contained in graphDat.csv and code in graphMeasures.Rmd lines 434–530 [112]. **D.** Boxplots display results of graph analysis for each region and timeset combination separately during encoding. Top, middle, and bottom panels display characteristic path length, weighted node strength, and unweighted node strength respectively. The colors of dots represent the region of each connection partner. Asterisks indicate significant (p < .0001) holm-corrected comparisons between HFB-aligned and image-aligned values within region. Notice that Hip and pPFC graphs are more connected when aligned to HFB peaks during successful encoding. This is true for the pPFC during failed encoding as well. This panel can be regenerated using data contained in graphDat.csv and code in graphMeasures.Rmd lines 111–328 [112]. **E.** Similar to D except for retrieval. This panel can be regenerated using data contained in graphDat.csv and code in graphMeasures.Rmd lines 111–328 [112].(PDF)

S11 Fig Example HFB peaks do not look like sharp wave ripples, but their mean does. **A-D** Line plots display individual HFB peak events. The raw timeseries is displayed in black and the timeseries bandpass filtered to between 70 and 150 Hz is displayed in purple. Panel ii displays a zoomed in time scale relative to panel i. Time is displayed on the *x* axis relative to the time point of HFB peak detection. The *y* axis displays the electrical potential measured in microvolts. All randomly chosen events are from hippocampal channels. Each event is recorded in a different participant. **E.** The line plot displays the mean of the raw hippocampal encoding timeseries aligned to the nearest trough of the HFB signal relative to the peak of the HFB power for hit and miss trials separately. Notice that although no individual events bear visual resemblance to classical sharp wave ripples, these mean events do. These panels can be regenerated using data contained in TF_hip_sub_HFB.mat and code in supFigure11.m [112].
(PDF)

S1 Table Participant demographics and key statistics. (PDF)

## Abbrevaitions

ACC: anterior cingulate cortex
dlPFC: dorsolateral prefrontal cortex
HFB: high-frequency broadband
Hip: hippocampus
ITPC: inter-trial phase clustering
iEEG: intracranial EEG
MTL: medial temporal lobe
PPC: pairwise phase consistency
PHG: parahippocampal gyrus
pPFC: polar prefrontal cortex
PFC: prefrontal cortex
ROIs: regions of interest
SWR: sharp-wave ripple
