# Supplementary material for: Episodic memory involves transient and sparse connectivity aligned to both internal and external events
Source: PLoS Biol. 2025 Nov 25;23(11):e3003481. doi: 10.1371/journal.pbio.3003481 (PMC12646405; doi:10.1371/journal.pbio.3003481)
Supplement: S1 Fig — A. Each line plot displays the mean time series of the HFB response across channels within different regions. For all panels, the x-axis displays time relative to image onset, and the y-axis displays HFB power in z-scored units. Encoding and retrieval data are plotted along the top and bottom rows, respectively. Orange and blue lines are the average time series of (subsequent) hit and miss trials, respectively. Vertical gray shaded regions indicate p < .05 for the difference between hit and miss after cluster correction. Successful encoding was associated with elevated HFB activity prior to image presentation in the dlPFC, and ~500 ms after image presentation and after the indoor/outdoor behavioral response in the pPFC. Successful retrieval was associated with elevated HFB activity prior to image presentation in the dlPFC, but lower HFB activity for failed memory late in the trial in both the dlPFC and pPFC. The PHG exhibited effects during both encoding and retrieval, but these were much later and smaller in magnitude than the visual response. Colored shaded regions indicate the standard error of the mean. Colored vertical dashed lines indicate mean reaction times for hit trials (blue) and miss trials (orange). This panel can be regenerated using data contained in the HFB_image folder and code in SupFigure1A.m [112]. B. Each grouped scatter plot displays the mean power of HFB peak for each channel grouped by region. Mean z-scored HFB power at the time of the HFB peak is displayed on the y-axis. Error bars display the 83% confidence interval around model estimates [113,114]. Linear mixed effects modeling of these data revealed main effects of encode/retrieve (χ2(1)=188,p<2e−16) and hit/miss (χ2(1)=11,p=.0008), and all four interaction terms: hit/miss by encode/retrieve (χ2(1)=6,=.016), hit/miss by region (χ2(1)=11,p=.02), encode/retrieve by region (χ2(1)=14,p=.007), and hit/miss by encode/retrieve by region (χ2(4)=20,p=.0005). Holm-corrected pairwise contrasts revea [file pbio.3003481.s001.pdf]

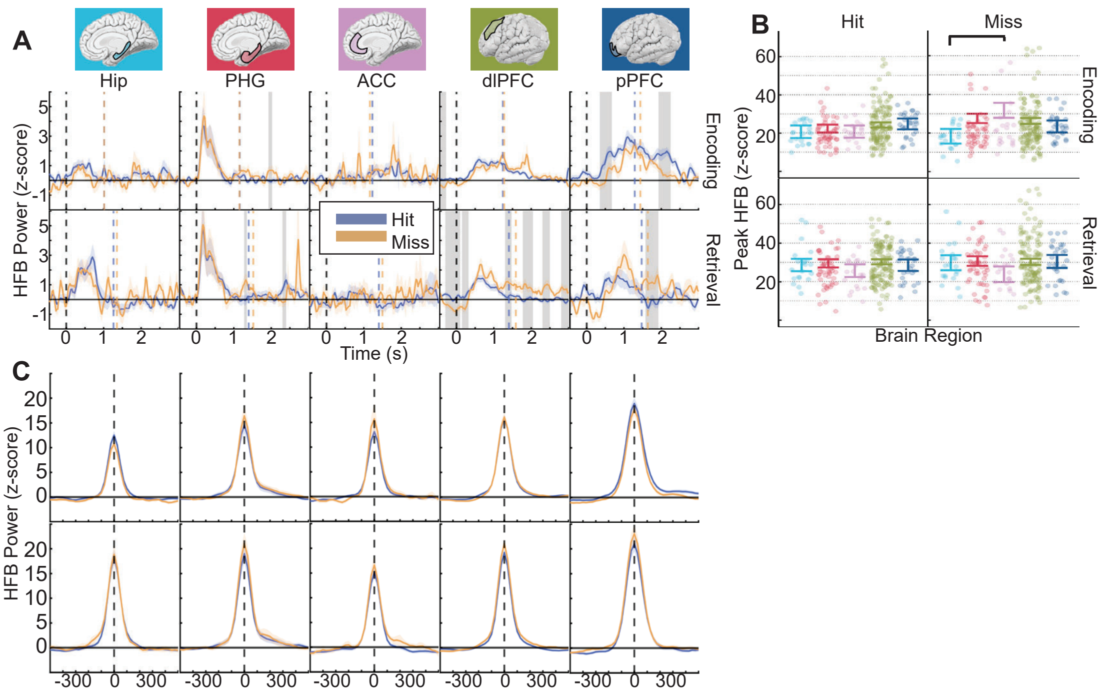

Supplemental Figure 1. HFB power changes aligned to image onset and HFB peak. A. Each line plot displays the mean time series of the HFB response across channels within different regions. For all panels, the x-axis displays time relative to image onset, and the y-axis displays HFB power in z-scored units. Encoding and retrieval data are plotted along the top and bottom rows, respectively. Orange and blue lines are the average time series of (subsequent) hit and miss trials, respectively. vertical gray shaded regions indicate p<.05 for the difference between hit and miss after cluster correction. Successful encoding was associated with elevated HFB activity prior to image presentation in the dlPFC, and ~500 ms after image presentation and after the indoor/outdoor behavioral response in the pPFC. Successful retrieval was associated with elevated HFB activity prior to image presentation in the dlPFC, but lower HFB activity for failed memory late in the trial in both the dlPFC and pPFC. The PHG exhibited effects during both encoding and retrieval, but these were much later and smaller in magnitude than the visual response. Colored shaded regions indicate the standard error of the mean. Colored vertical dashed lines indicate mean reaction times for hit trials (blue) and miss trials (orange). B. Each grouped scatter plot displays the mean power of HFB peak for each channel grouped by region. Mean z-scored HFB power at the time of the HFB peak is displayed on the y axis. Error bars display the 83% confidence interval around model estimates (112,113). Linear mixed effects modeling of these data revealed main effects of encode/retrieve (2(1)=188, p<2e-16) and hit/miss (2(1)=11, p=.0008), and all four interaction terms: hit/miss by encode/retrieve (2(1)=6, =.016), hit/miss by region (2(1)=11, p=.02), encode/retrieve by region (2(1)=14, p=.007), and hit/miss by encode/retrieve by region (2(4)=20, p=.0005). Holm-corrected pairwise contrasts revealed that in the PHG, Hip, dlPFC, and pPFC, the peak HFB power was higher during retrieval than encoding. Mnemonic effects were evident in the ACC and PHG, with higher peak power during failed encoding than successful encoding. C. Mean HFB power time series using the same conventions as in A except with time centered around the latency of the HFB peak. Both panels display data from the encoding phase of the experiment, with ACC data displayed on top and PHG data below. Note that although the image-locked mean HFB activity in the ACC appeared flat (panel A), its peak activity was no smaller than in any other region.
