# Supplementary material for: Episodic memory involves transient and sparse connectivity aligned to both internal and external events
Source: PLoS Biol. 2025 Nov 25;23(11):e3003481. doi: 10.1371/journal.pbio.3003481 (PMC12646405; doi:10.1371/journal.pbio.3003481)
Supplement: S2 Fig — A. Each grouped scatter plot displays the mean time of peak HFB latency for each channel grouped by region. Time relative to image onset is displayed in milliseconds on the y-axis. Error bars display the 83% confidence interval around model estimates [113,114]. B. Similar to A except a subset of trials was selected such that reaction time was matched at the individual trial level. C. Similar to B, except that a subset of trials was selected, such that reaction time was matched at the individual trial level, and normalized time was used. D. The asynchrony of HFB latency within trial is displayed for pairs of channels recorded simultaneously within individual participants. The four groups of figures represent the behavioral conditions indicated by the large font labels in the left and top margins. The x-axis shows the difference in timing of the HFB peak observed on single trials at pairs of simultaneously recorded channels. Positive values correspond to the brain region indicated at the top of the column that had an earlier HFB peak latency. Negative values correspond to the brain region indicated at the side of the column that had an earlier HFB peak latency. The y-axis shows the proportion of trials observed to have a given peak latency asynchrony. The color of the line represents the brain region with the earlier latency (as judged by proportion of trials). The dashed line shows the latency asynchronies observed when region identity is shuffled prior to calculating latency asynchrony. The percent of trials where the row region was the leader is indicated in the upper left of each plot. The percent of trials where the column region was the leader is indicated in the upper right. Note, these values do not sum to 100% because ties were discounted. The pairwise contrasts observed here recapitulate the order observed in Fig 2D of the main text. All panels can be regenerated using data contained in trialLatDat_RTfix.csv and code in Latency_LME_modeling.Rmd lines 736–824 [file pbio.3003481.s002.pdf]

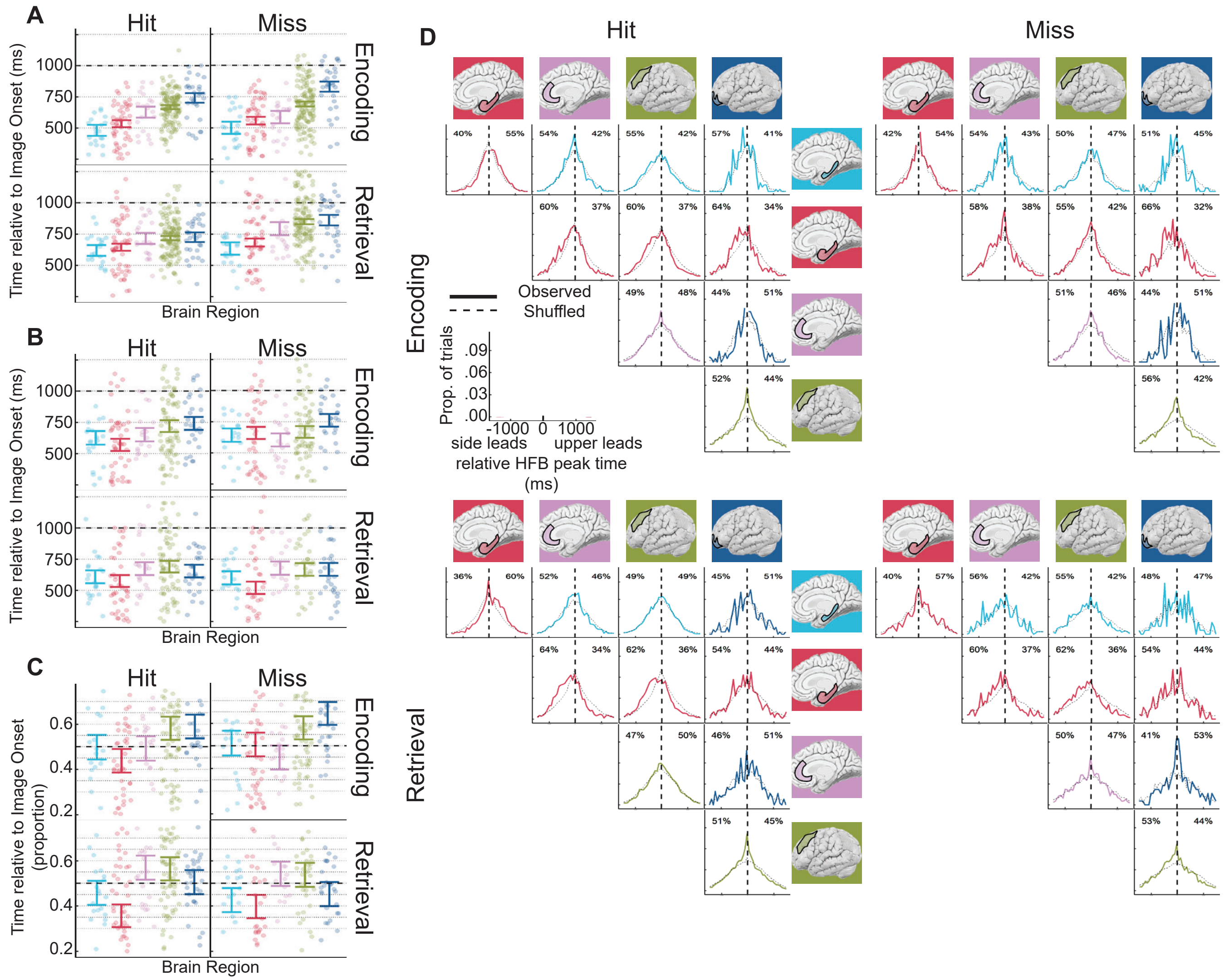

Supplemental Figure 2. Timing of HFB peak latency can be measured in several ways, but all reinforce the same general interpretation. A. Each grouped scatter plot displays the mean time of peak HFB latency for each channel grouped by region. Time relative to image onset is displayed in milliseconds on the y axis. Error bars display the 83% confidence interval around model estimates (112,113). B. Similar to A except a subset of trials was selected such that reaction time was matched at the individual trial level. C. Similar to B, except that a subset of trials was selected, such that reaction time was matched at the individual trial level, and normalized time was used. D. The asynchrony of HFB latency within trial is displayed for pairs of channels recorded simultaneously within individual participants. The four groups of figures represent the behavioral conditions indicated by the large font labels in the left and top margins. The x axis shows the difference in timing of the HFB peak observed on single trials at pairs of simultaneously recorded channels. Positive values correspond to the brain region indicated at the top of the column having had an earlier HFB peak latency. Negative values correspond to the brain region indicated at the side of the column having had an earlier HFB peak latency. The y axis shows the proportion of trials observed to have a given peak latency asynchrony. The color of the line represents the brain region with the earlier latency (as judged by proportion of trials). The dashed line shows the latency asynchronies observed when region identity is shuffled prior to calculating latency asynchrony. The percent of trials where the row region was the leader is indicated in the upper left of each plot. The percent of trials where the column region was the leader is indicated in the upper right. Note, these values do not sum to 100% because ties were discounted. The pairwise contrasts observed here recapitulate the order observed in Figure 2D of the main text.
