# Supplementary material for: Episodic memory involves transient and sparse connectivity aligned to both internal and external events
Source: PLoS Biol. 2025 Nov 25;23(11):e3003481. doi: 10.1371/journal.pbio.3003481 (PMC12646405; doi:10.1371/journal.pbio.3003481)
Supplement: S3 Fig — A. heatmaps display frequency in Hertz on the y axis and time in milliseconds relative to HFB peak on the x axis. Color indicates power z-scored within frequency. The top row reflects data collected during subsequent hit trials. The bottom row reflects data collected during subsequent miss trials. B. Similar to A except for retrieval. C. To facilitate comparison between hit and miss trials, line plots display power spectra at the time point of the HFB peak. For all panels, the x-axis displays frequency, and the y-axis displays power in z-scored units. Encoding and retrieval data are plotted along the top and bottom rows, respectively. Orange and blue lines are the average spectra of (subsequent) hit and miss trials, respectively. Vertical gray shaded regions indicate p < .05 for the difference between hit and miss spectra after cluster correction. Colored shaded regions indicate the standard error of the mean. Successful encoding elicited greater power in the Hip between 2.0 and 2.9 Hz. Failed retrieval elicited greater power in the PHG between 4.4 and 15 Hz. Although other ROIs did not exhibit hit/miss differences, power spectra nevertheless appeared different across regions and between encoding and retrieval. These differences were explored using linear mixed effects modeling of power as a function of frequency (2–80 Hz; modeled with 8 splines), region, and encoding/retrieval. All interactions between fixed effects were significant (χ2(4−32)>66,maximump<2e−10). Several factors may explain these interactions. First, an interaction between frequency and region may have been driven by regional variations in the frequency of maximum power in the lower range (2–20 Hz; S1 Table). Second, an interaction between encode/retrieve and region may have been driven by higher power in the Hip during retrieval than during encoding. Third, a three-way interaction may have been driven by secondary power increases between 24 and 32 Hz in the Hip, ACC, and dlPFC during retrieval (S1 [file pbio.3003481.s003.pdf]

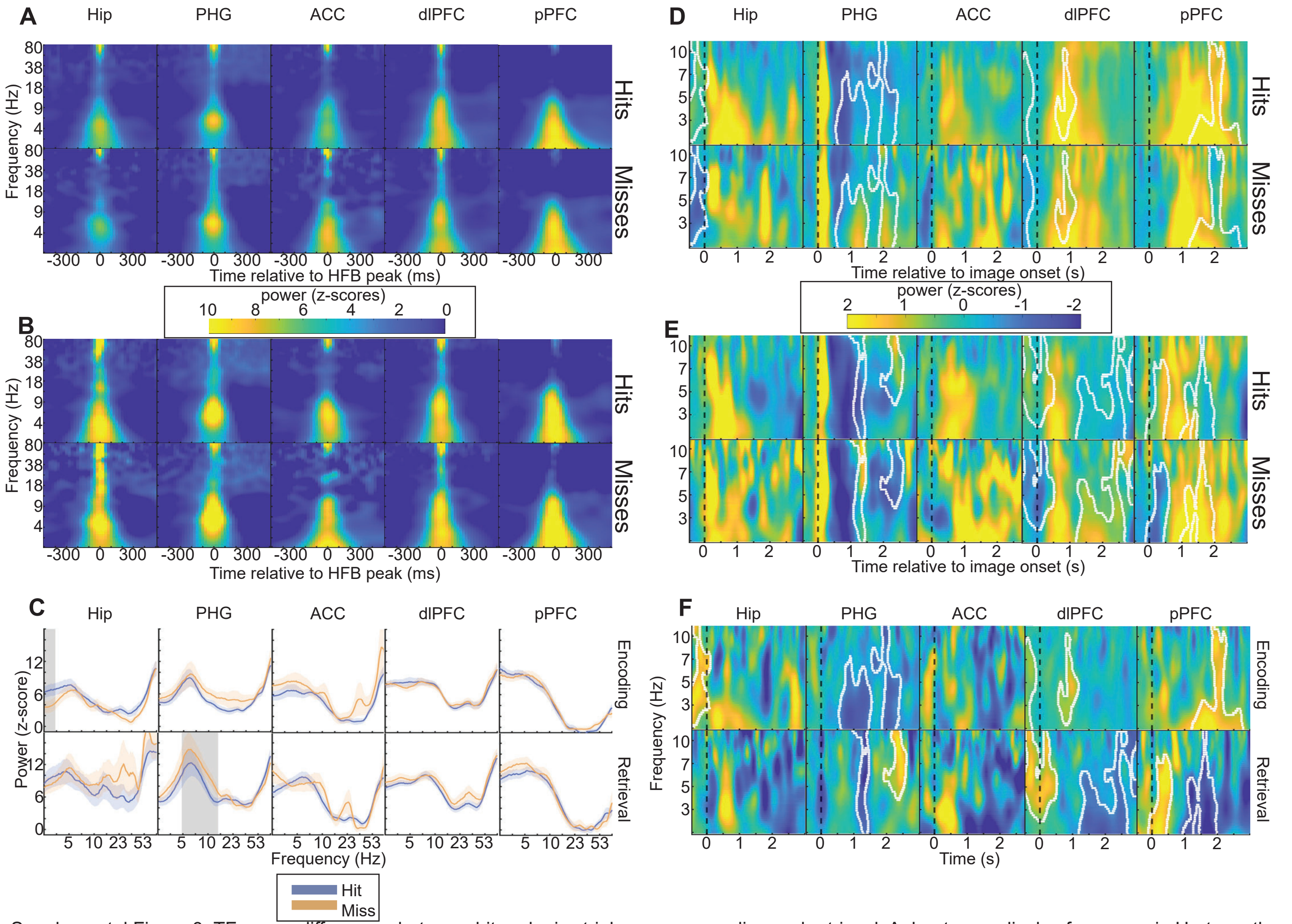

Supplemental Figure 3. TF power differences between hit and miss trials across encoding and retrieval. A. heatmaps display frequency in Hertz on the y axis and time in milliseconds relative to HFB peak on the x axis. Color indicates power z-scored within frequency. The top row reflects data collected during subsequent hit trials. The bottom row reflects data collected during subsequent miss trials. B. Similar to A except for retrieval. C. To facilitate comparison between hit and miss trials, line plots display power spectra at the time point of the HFB peak. For all panels, the x-axis displays frequency, and the y-axis displays power in z-scored units. Encoding and retrieval data are plotted along the top and bottom rows, respectively. Orange and blue lines are the average spectra of (subsequent) hit and miss trials, respectively. vertical gray shaded regions indicate p<.05 for the difference between hit and miss spectra after cluster correction. Colored shaded regions indicate the standard error of the mean. Successful encoding elicited greater power in the Hip between 2.0 and 2.9 Hz. Failed retrieval elicited greater power in the PHG between 4.4 Hz and 15 Hz. Although other ROIs did not exhibit hit/miss differences, power spectra nevertheless appeared different across regions and between encoding and retrieval. These differences were explored using linear mixed effects modeling of power as a function of frequency (2-80 Hz; modeled with 8 splines), region, and encoding/retrieval. All interactions between fixed effects were significant (2(4-32)>66,maximum p <2e-10). Several factors may explain these interactions. First, an interaction between frequency and region may have been driven by regional variations in the frequency of maximum power in the lower range (2-20 Hz; Supplemental Table 1). Second, an interaction between encode/retrieve and region may have been driven by higher power in the Hip during retrieval than during encoding. Third, a three-way interaction may have been driven by secondary power increases between 24 and 32 Hz in the Hip, ACC, and dlPFC during retrieval (Supplemental Table 1). These results emphasize that HFB peaks constituted physiological events, and not statistically extreme values. D. Similar to A, except time on the x axis is represented in seconds relative to the image onset. E. Similar to D, except for retrieval. F. Heatmaps display the mean difference in z-scored power between hit and miss trials. For all panels, the x-axis displays time relative to image onset, and the y-axis displays frequency. White outlined areas indicate p<.05 for the difference between hit and miss power after cluster correction. In the Hip and dlPFC, there were positive subsequent memory effects in 2 to 10 Hz power just before and during image onset (-450 to 100 ms). In the PHG, there was a negative memory effect in 2 to 10 Hz later after image onset at both encoding (550 to 1550 ms) and retrieval (925 to 1450 ms). These decreases partially overlapped with the visual response observed in the PHG's HFB power (Supplemental Figure 1A). Finally, the pPFC exhibited a late (1225 to 2775 ms) positive subsequent memory effect during encoding across 2 to 10 Hz. At retrieval, the pPFC exhibited a much earlier (-100 to 600 ms) positive memory effect and a later (950 to 1975 ms) negative memory effect.
