# Supplementary material for: Episodic memory involves transient and sparse connectivity aligned to both internal and external events
Source: PLoS Biol. 2025 Nov 25;23(11):e3003481. doi: 10.1371/journal.pbio.3003481 (PMC12646405; doi:10.1371/journal.pbio.3003481)
Supplement: S4 Fig — A. Histograms display the proportion of channels on the y axis and the phase of either the 3 Hz (left columns) or 6.5 Hz (right columns) component of the signal. For each channel, the mean circular phase was calculated across trials at the time point of the HFB peak for hit (blue) and miss (orange) trials separately. The superimposed sin wave on each plot indicates which phase values correspond to the peak, trough, or intermediate positions in the oscillation. The dashed-dotted lines indicate uniform distribution. The top row of each panel displays encoding data. The bottom row displays retrieval data. Each panel displays data for a different region. Notice that the Hip exhibited a preference for HFB activity to occur at the trough of the 3 Hz oscillation during successful memory trials at both encoding and retrieval, but the PHG exhibited a preference for the peak. This panel can be regenerated using data contained in the TFphase_HFB folder and code in Figure3A_supFigure4A.m [112]. B. Similar to A, but mean phase was measured at the time point of maximal ITPC relative to image onset. Notice that phase preferences evident in MTL regions in C appear weaker when measured relative to image onset. This panel can be regenerated using data contained in the TFphase_image folder and code in Figure3B_supFigure4B.m [112]. (PDF) [file pbio.3003481.s004.pdf]

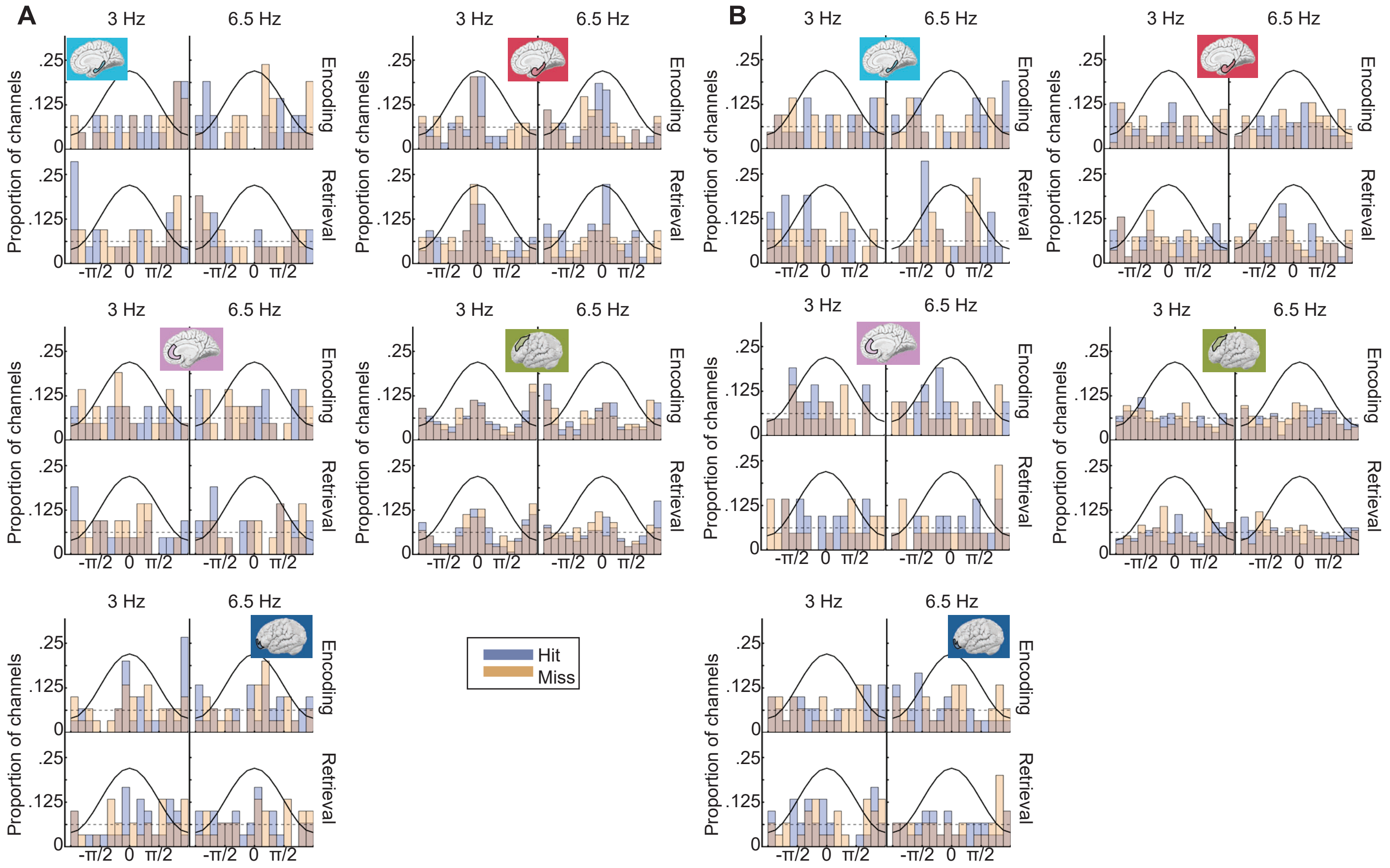

Supplemental Figure 4. Distributions of phase preference of phase clustering within channels. A. Histograms display the proportion of channels on the y axis and the phase of either the 3 Hz (left columns) or 6.5 Hz (right columns) component of the signal. For each channel, the mean circular phase was calculated across trials at the time point of the HFB peak for hit (blue) and miss (orange) trials separately. The superimposed sin wave on each plot indicates which phase values correspond to the peak, trough, or intermediate positions in the oscillation. The dashed-dotted lines indicate uniform distribution. The top row of each panel displays encoding data. The bottom row displays retrieval data. Each panel displays data for a different region. Notice that the Hip exhibited a preference for HFB activity to occur at the trough of the 3 Hz oscillation during successful memory trials at both encoding and retrieval, but the PHG exhibited a preference for the peak. B. Similar to A, but mean phase was measured at the time point of maximal ITPC relative to image onset. Notice that phase preferences evident in MTL regions in C appear weaker when measured relative to image onset.
