# Supplementary material for: Episodic memory involves transient and sparse connectivity aligned to both internal and external events
Source: PLoS Biol. 2025 Nov 25;23(11):e3003481. doi: 10.1371/journal.pbio.3003481 (PMC12646405; doi:10.1371/journal.pbio.3003481)
Supplement: S5 Fig — Plots A and B correspond to main text Fig 4E and 4F, respectively. However, while the schematics in Fig 4E and 4F display only connections that exhibited increased strength for hit trials, this figure displays only connections that exhibited decreased connectivity strength for hit trials relative to miss trials. Note that far fewer of these negative connections were detected than the positive connections shown in Fig 4. These panels can be regenerated using data contained in HFBConnections.mat and imageConnections.mat and code in Figure4EF.m [112]. (PDF) [file pbio.3003481.s005.pdf]

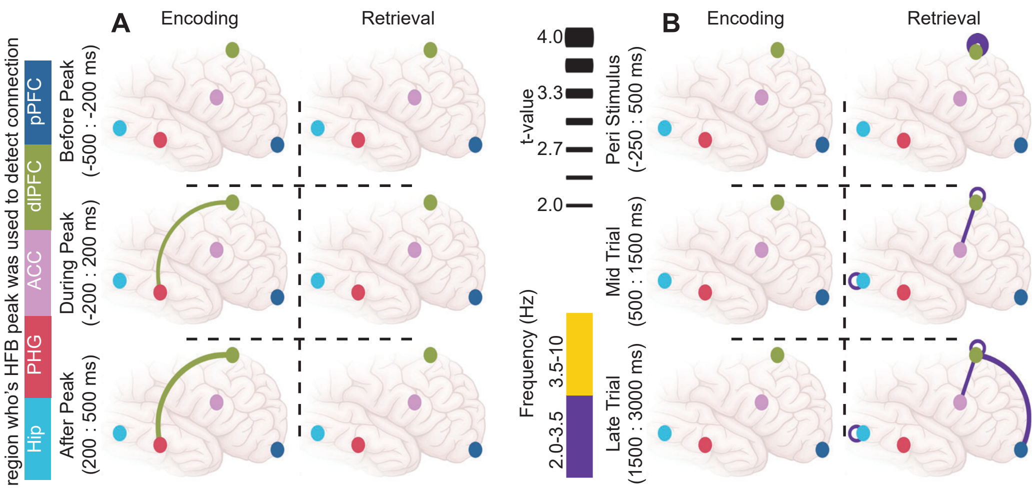


Supplemental Figure 5. Negative mnemonic connections. Plots A and B correspond to main text Figure 4 E and F, respectively. However, while the schematics in Figure 4 E and F display only connections that exhibited increased strength for hit trials, this figure displays only connections that exhibited decreased connectivity strength for hit trials relative to miss trials. Note that far fewer of these negative connections were detected than the positive connections shown in Figure 4.
