# Supplementary material for: Episodic memory involves transient and sparse connectivity aligned to both internal and external events
Source: PLoS Biol. 2025 Nov 25;23(11):e3003481. doi: 10.1371/journal.pbio.3003481 (PMC12646405; doi:10.1371/journal.pbio.3003481)
Supplement: S7 Fig — Analysis aligned to HFB peak of the region indicated by the row position of each subplot. In all panels, subplots have time relative to HFB peak (t = 0) on the X-axis and frequency on the Y-axis. The color scale indicates the strength of connectivity between the regions indicated by the row and column of the subplot. A. Connectivity during subsequent hit trials measured using pairwise phase consistency. B. Connectivity during subsequent miss trials measured using pairwise phase consistency. C. The difference between hit and miss trials. Warm colors indicate stronger connectivity during hit trials. Color scale represents t-values. White outlines indicate cluster-corrected significant differences between hit and miss trials. These panels can be regenerated using data contained in the connectionDat folder and code in SupFigure6_7_8_9_10A.m [112]. (PDF) [file pbio.3003481.s007.pdf]

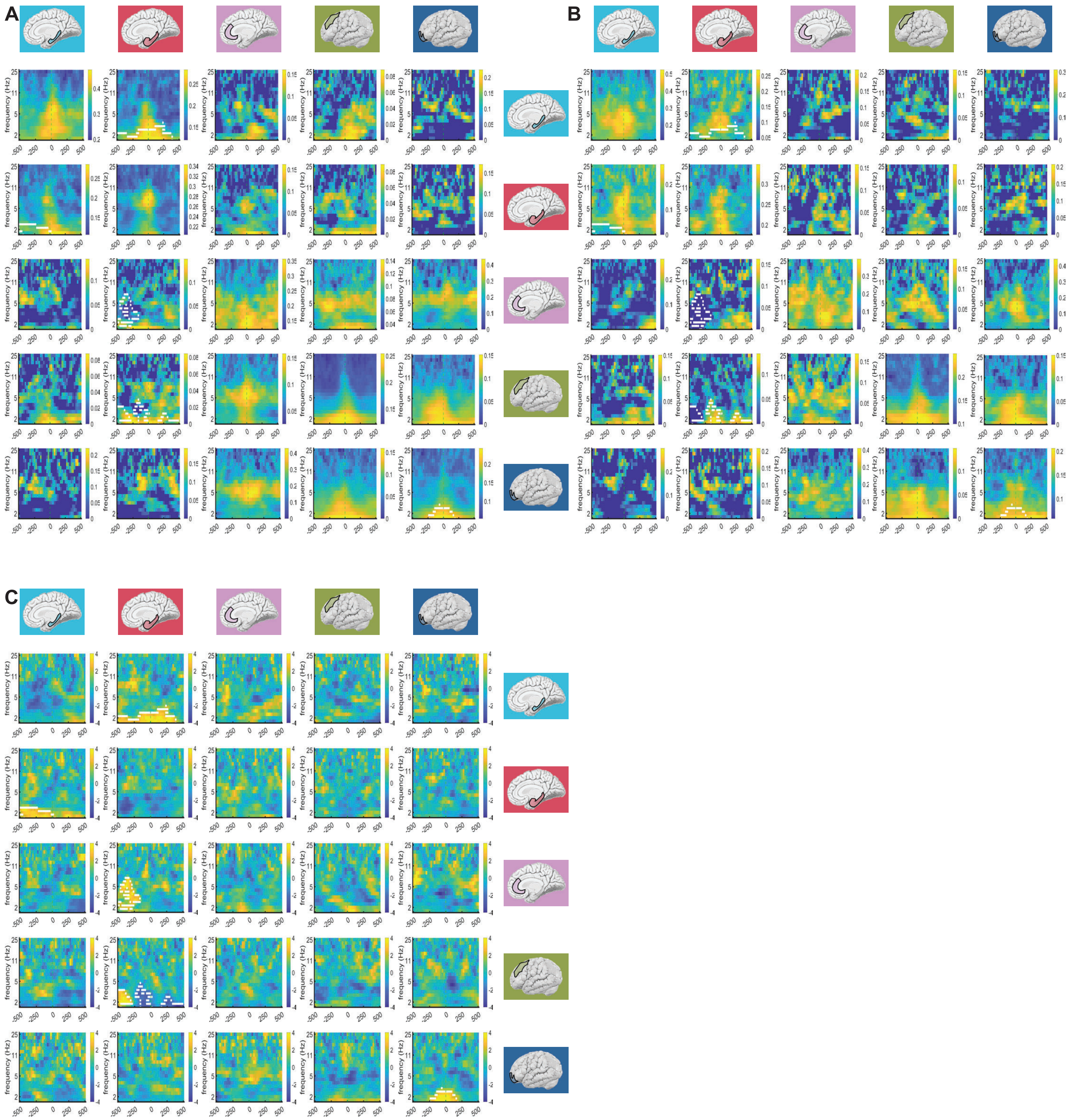

Supplemental Figure 7. Inter-regional connectivity changes associated with memory during encoding. Analysis aligned to HFB peak of the region indicated by the row position of each subplot. In all panels, subplots have time relative to HFB peak (t=0) on the X-axis and frequency on the Y-axis. The color scale indicates the strength of connectivity between the regions indicated by the row and column of the subplot. A. Connectivity during subsequent hit trials measured using pairwise phase consistency. B. Connectivity during subsequent miss trials measured using pairwise phase consistency. C. The difference between hit and miss trials. Warm colors indicate stronger connectivity during hit trials. Color scale represents t-values. White outlines indicate cluster-corrected significant differences between hit and miss trials.
