# Supplementary material for: Episodic memory involves transient and sparse connectivity aligned to both internal and external events
Source: PLoS Biol. 2025 Nov 25;23(11):e3003481. doi: 10.1371/journal.pbio.3003481 (PMC12646405; doi:10.1371/journal.pbio.3003481)
Supplement: S10 Fig — A. Box plot display connectivity between the ACC and Hip for all channel pairs that spanned the two regions (open blue circles) and participants (red circles) averaged across the same time and frequency windows as in Fig 5A.i and 5A.ii. Note that although miss trials were characterized by more densely connected overall graphs (Fig 5A.ii), connectivity between the ACC and Hip was weaker during miss trials for all participants. This panel can be regenerated using data contained in the connectionDat folder and code in SupFigure6_7_8_9_10A.m [112]. B. The histogram displays the strength of all connections for the representative participant shown in Fig 5A averaged across the long temporal epoch (corresponding to Fig 5A.iii and 5A.iv). Note that with lower variance in the hit distribution, a higher central tendency in the hit distribution, and similar representation at very strong connectivity strengths between hit and miss trials (see inset), a z-scored analysis would replicate prior findings of stronger connectivity for hit trials [47]. Overall, for this example participant, 52% of all possible connections exhibited greater connectivity values for successful encoding over failed encoding, and the corresponding value was 54% for retrieval trails. It is interesting that overall connectivity was stronger during hit trials when examined over the longer temporal window. This panel can be regenerated using data contained in hip_acc_ret_HFB_2_21.mat and code in Figure5A_supFigure10B.m [112]. C. Same as in Fig 5B but with data sorted by whether significant connections were detected in HFB-aligned or image-aligned analyses. There was a modest effect such that graphs exhibited more connectivity for HFB-locked analyses than image-locked: shorter characteristic path length, increased weighted strength, and increased unweighted strength; χ2(1)>12,maximump<.0005. This panel can be regenerated using data contained in graphDat.csv and code in graphMeasures.Rmd lines 434–530 [112]. D. [file pbio.3003481.s010.pdf]

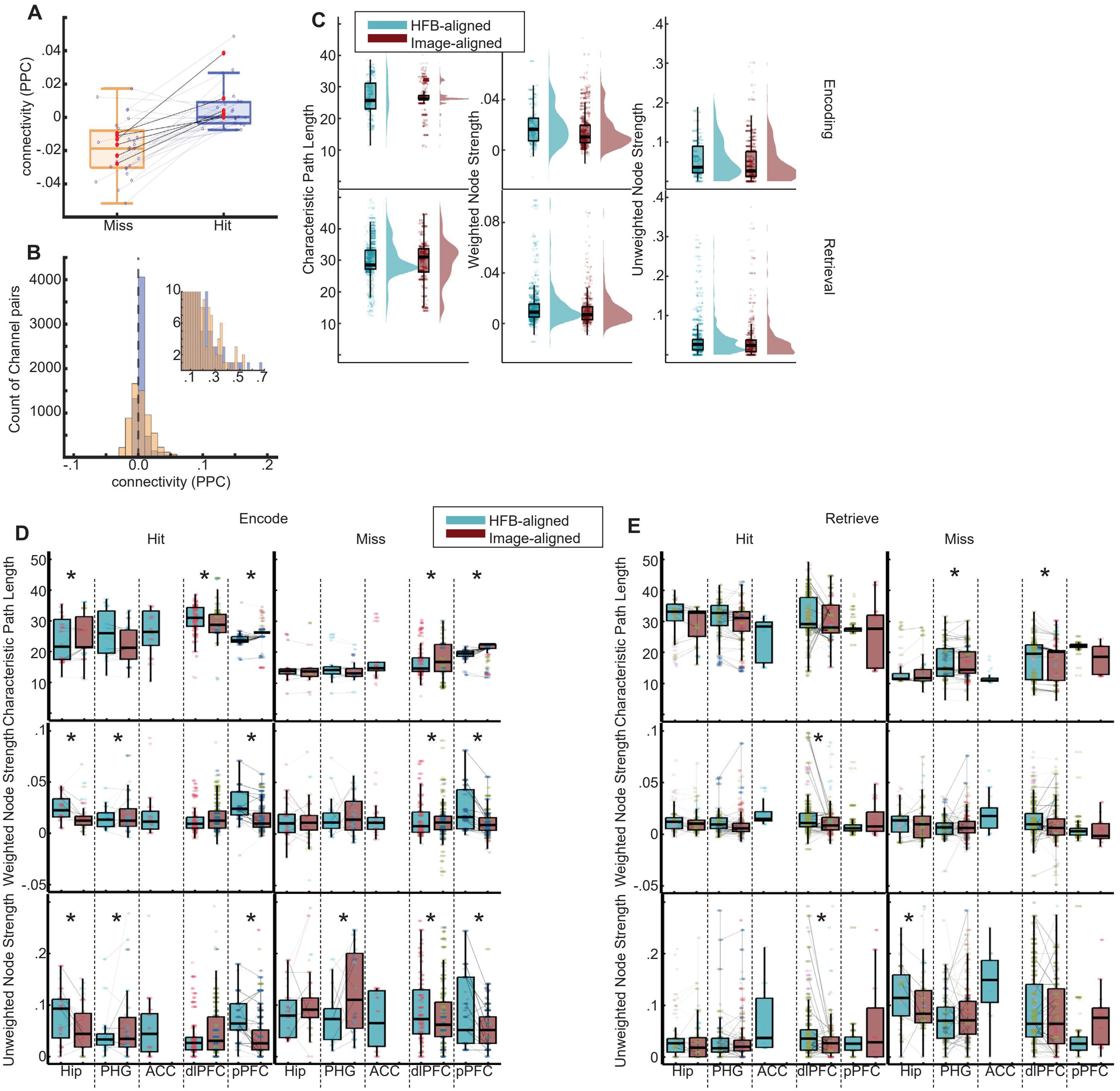

Supplemental Figure 10. Additional graph analysis results. A. Box plot display connectivity between the ACC and Hip for all channel pairs that spanned the two regions (open blue circles) and participants (red circles) averaged across the same time and frequency windows as in Figure 5A.i and A.ii. Note that although miss trials were characterized by more densely connected overall graphs (A.ii), connectivity between the ACC and Hip was weaker during miss trials for all participants. B. The histogram displays the strength of all connections for the representative participant shown in Figure 5A averaged across the long temporal epoch (corresponding to Figure A.iii and A.iv). Note that with lower variance in the hit distribution, a higher central tendency in the hit distribution, and similar representation at very strong connectivity strengths between hit and miss trials (see inset), a z-scored analysis would replicate prior findings of stronger connectivity for hit trials (47). Overall, for this example participant, 52% of all possible connections exhibited greater connectivity values for successful encoding over failed encoding, and the corresponding value was 54% for retrieval trails. It is interesting that overall connectivity was stronger during hit trials when examined over the longer temporal window. C. Same as in Figure 5B but with data sorted by whether significant connections were detected in HFB-aligned or image-aligned analyses. There was a modest effect such that graphs exhibited more connectivity for HFB-locked analyses than image-locked: shorter characteristic path length, increased weighted strength, and increased unweighted strength; 2(1)>12,maximum $p < .0005$. D. Boxplots display results of graph analysis for each region and timeset combination separately during encoding. Top, middle, and bottom panels display characteristic path length, weighted node strength, and unweighted node strength respectively. The colors of dots represent the region of each connection partner. Asterisks indicate significant ($p<.0001$) holm-corrected comparisons between HFB-aligned and image-aligned values within region. Notice that Hip and pPFC graphs are more connected when aligned to HFB peaks during successful encoding. This is true for the pPFC during failed encoding as well. E. Similar to D except for retrieval.
