# Supplementary material for: Episodic memory involves transient and sparse connectivity aligned to both internal and external events
Source: PLoS Biol. 2025 Nov 25;23(11):e3003481. doi: 10.1371/journal.pbio.3003481 (PMC12646405; doi:10.1371/journal.pbio.3003481)
Supplement: S11 Fig — A-D Line plots display individual HFB peak events. The raw timeseries is displayed in black and the timeseries bandpass filtered to between 70 and 150 Hz is displayed in purple. Panel ii displays a zoomed in time scale relative to panel i. Time is displayed on the x axis relative to the time point of HFB peak detection. The y axis displays the electrical potential measured in microvolts. All randomly chosen events are from hippocampal channels. Each event is recorded in a different participant. E. The line plot displays the mean of the raw hippocampal encoding timeseries aligned to the nearest trough of the HFB signal relative to the peak of the HFB power for hit and miss trials separately. Notice that although no individual events bear visual resemblance to classical sharp wave ripples, these mean events do. These panels can be regenerated using data contained in TF_hip_sub_HFB.mat and code in supFigure11.m [112]. (PDF) [file pbio.3003481.s011.pdf]

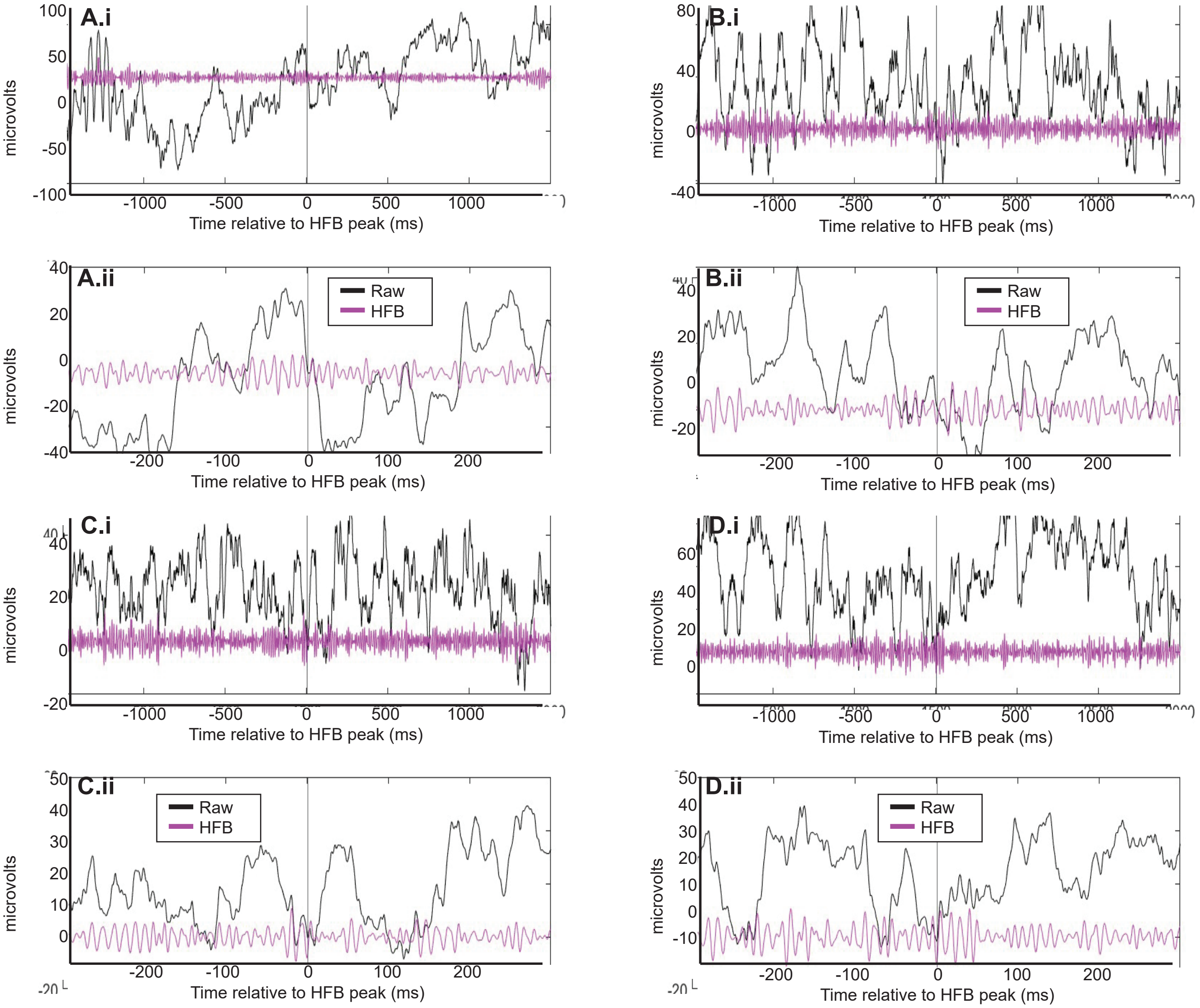

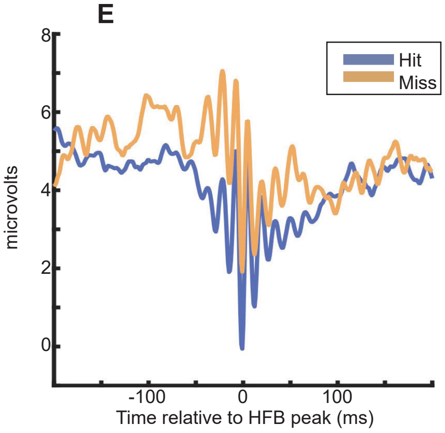

Supplemental Figure 11. Example HFB peaks do not look like sharp wave ripples, but their mean does. A-D Line plots display individual HFB peak events. The raw timeseries is displayed in black and the timeseries bandpass filtered to between 70 and 150 Hz is displayed in purple. Panel ii displays a zoomed in time scale relative to panel i. Time is displayed on the x axis relative to the time point of HFB peak detection. The y axis displays the electrical potential measured in microvolts. All randomly chosen events are from hippocampal channels. Each event is recorded in a different participant. E. The line plot displays the mean of the raw hippocampal encoding timeseries aligned to the nearest trough of the HFB signal relative to the peak of the HFB power for hit and miss trials separately. Notice that although no individual events bear visual resemblance to classical sharp wave ripples, these mean events do.
