# Supplementary material for: Episodic memory involves transient and sparse connectivity aligned to both internal and external events
Source: PLoS Biol. 2025 Nov 25;23(11):e3003481. doi: 10.1371/journal.pbio.3003481 (PMC12646405; doi:10.1371/journal.pbio.3003481)
Supplement: S1 Table — (PDF) [file pbio.3003481.s012.pdf]

region	total channels	reactive channels	n (females)	age	memory accuracy	memory d'	hit trial count	miss trial count	ENCODING low freq peak	ENCODING med. freq peak	ENCODING max ITPC	RETRIEVAL low freq peak	RETRIEVAL med. freq peak	RETRIEVAL max ITPC
Hip	40	21	9 (3)	19.01 (4.7)	.50 (.22)	1.54 (.80)	59.8 (36.0)	24.3 (13.3)	4.9	-	6.1	4.2	27.1	-
PHG	79	54	23 (9)	18.4 (4.7)	.51 (.21)	1.54 (.73)	50.3 (24.5)	21.2 (12.4)	5.7	-	20.9	5.9	-	9.5
ACC	44	21	11 (3)	19.4 (4.8)	.48 (.21)	1.54 (.71)	52.1 (37.9)	22.8 (10.8)	4.1	-	5.9	5.3	24.3	-
dlPFC	247	133	27 (9)	18.7 (4.8)	.50 (.22)	1.53 (.76)	51.6 (28.2)	21.0 (12.6)	6.6	-	19.4	7.6	31.5	19.4
pPFC	35	30	8 (3)	20.2 (4.32)	.40 (.19)	1.16 (.63)	41.5 (10.5)	21.6 (6.4)	2	-	6.1	4.9	-	-

Supplemental Table 1. Participant demographics and key statistics. Age, memory accuracy, d', and trial count columns are formatted as mean (SD).
